# GenAI synthesis of histopathological images from Raman imaging for intraoperative tongue squamous cell carcinoma assessment

**DOI:** 10.1038/s41368-025-00346-y

**Published:** 2025-01-26

**Authors:** Bing Yan, Zhining Wen, Lili Xue, Tianyi Wang, Zhichao Liu, Wulin Long, Yi Li, Runyu Jing

**Affiliations:** 1 State Key Laboratory of Oral Diseases & National Center for Stomatology & National Clinical Research Center for Oral Diseases & Department of Head and Neck Oncology Surgery, West China Hospital of Stomatology, Sichuan University, Chengdu, China; 2 College of Chemistry, Sichuan University, Chengdu, China; 3 Department of Stomatology, The first affiliated hospital of Xiamen University, Xiamen, China; 4 Nonclinical Drug Safety, Boehringer Ingelheim Pharmaceuticals, Inc., Ridgefield, CT USA; 5 School of Cyber Science and Engineering, Sichuan University, Chengdu, China; 6 Present Address: School of Mathematics and Big Data, Guizhou Education University, Guiyang, China

**Keywords:** Oral cancer, Cancer imaging

## Abstract

The presence of a positive deep surgical margin in tongue squamous cell carcinoma (TSCC) significantly elevates the risk of local recurrence. Therefore, a prompt and precise intraoperative assessment of margin status is imperative to ensure thorough tumor resection. In this study, we integrate Raman imaging technology with an artificial intelligence (AI) generative model, proposing an innovative approach for intraoperative margin status diagnosis. This method utilizes Raman imaging to swiftly and non-invasively capture tissue Raman images, which are then transformed into hematoxylin-eosin (H&E)-stained histopathological images using an AI generative model for histopathological diagnosis. The generated H&E-stained images clearly illustrate the tissue’s pathological conditions. Independently reviewed by three pathologists, the overall diagnostic accuracy for distinguishing between tumor tissue and normal muscle tissue reaches 86.7%. Notably, it outperforms current clinical practices, especially in TSCC with positive lymph node metastasis or moderately differentiated grades. This advancement highlights the potential of AI-enhanced Raman imaging to significantly improve intraoperative assessments and surgical margin evaluations, promising a versatile diagnostic tool beyond TSCC.

## Introduction

The tongue is a prevalent site for oral squamous cell carcinoma (OSCC), with tongue squamous cell carcinoma (TSCC) constituting approximately half of all OSCC cases.^1–3^ Surgical resection currently stands as the primary choice for TSCC treatment, with the goal of achieving extensive excision to eliminate residual cancer cells. A positive surgical margin, indicating the lingering cancer cells, may increase the risk of local recurrence by 90%, contributing to a substantial 90% increase in overall mortality rates at five years due to potential systemic diseases and associated health issues.^4^ Physicians grapple with the delicate balance of ensuring a sufficient free surgical margin to prevent cancer cell remnants while minimizing damage to normal tissues and functions during surgery. Extensive resection can significantly impact aspects of tongue function, such as speech, taste, and swallowing, thereby compromising overall quality of life.^5–7^ Therefore, the analysis of the deep surgical margin, which involves determining the depth of tumor infiltration into biological tissues, is crucial for evaluating the adequacy of tumor excision and ensuring a negative margin to prevent the persistence of cancer cells. Nevertheless, intraoperative determination of the deep surgical margin is difficult due to the unavailability of indicators commonly used for assessing deep tumor infiltration, such as lymphovascular invasion, through visual inspection or palpation.^4,8^ Previous studies have indicated a higher positivity rate for the deep surgical margin compared to the peripheral visible mucosal margins.^4,9,10^ Consequently, surgical margin analysis, particularly intraoperative analysis of the deep surgical margin, is imperative.^11–15^

Presently, intraoperative fresh frozen sectioning (FFS) is employed as the standard method for assessing tumor margins. Intraoperative FFS can generate hematoxylin-eosin (H&E)-stained images for assessing tissue pathology, guiding surgeons in determining the necessity for further re-resection. However, the impediments, encompassing issues such as tissue distortion during sampling, time constraints on tissue staining, and the potential for misinterpretation under the microscope, persistently compromise the accuracy of intraoperative frozen section interpretation.^4^ High-quality H&E images can be obtained through standard postoperative histopathology specimen processing, but this process is meticulous and time-intensive, involving sequential steps such as fixation, dehydration, and staining, typically taking 12 to 48 h. Ozyoruk et al. introduced a generative adversarial network (GAN)-based model designed to transform cryosectioned images into formalin-fixed and paraffin-embedded (FFPE)-style images, aiming to enhance the efficiency of intraoperative diagnosis.^16^ Nevertheless, this approach still necessitates the freezing and sectioning of tissue samples for histopathological imaging. Consequently, the effective intraoperative acquisition of H&E-stained histopathology images for surgeons to make decisions remains a clinical challenge.^17,18^ To date, intraoperative surgical margin analysis of TSCC has been inadequately addressed, lacking a rapid, non-invasive, and in vivo detection method.

Previous studies have explored the potential applications of Raman spectroscopy in cancer pathology, with a primary focus on tissue classification and subtype identification.^19–29^ In recent years, in vivo Raman spectroscopy has been investigated for its application in the diagnosis of bladder cancer and the detection of colon cancer.^30–36^ The clinical advantages of Raman imaging encompass its capacity to generate detailed chemical maps, unveiling spatial distributions of specific components. This includes mapping cell structures and identifying disease markers, closely correlated with cytopathology.^37^ Additionally, Raman imaging can be conducted in a label-free manner, obviating the need for exogenous contrast agents. This positions it as a promising alternative for swiftly scanning tissue samples in vivo and producing real-time digital images. Here, we propose an innovative approach integrating Raman imaging technology with an artificial intelligence (AI) generative model to rapidly generate tissue H&E images. Using our AI model, we successfully converted Raman images into H&E images with distinct histological features for normal tissue and TSCC. Combining Raman imaging with the diffusion-based AI model demonstrates significant potential as an efficient and in vivo alternative tool for intraoperative surgical margin analysis of TSCC.

## Results

### Comparison of histopathology imaging and Raman imaging procedures

**Fig. 1:**
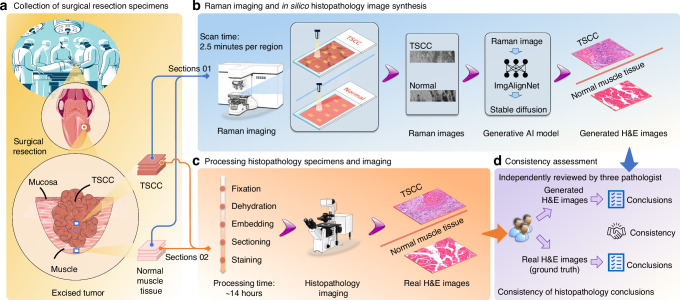
Overview of the study design. **a** Tissue sample collection. Tongue squamous cell carcinoma (TSCC) and corresponding normal muscle tissue samples were obtained from surgical resection specimens in the operating room. **b** Raman imaging and in silico histopathology image synthesis. After cryosectioning, TSCC and normal tissues were directly placed on the Raman imaging instrument for scanning. This process yielded corresponding Raman images, with a scanning time of 2.5 min for each region. Utilizing AI generated model, the H&E images can be generated based on the Raman images. **c** Histopathological processing and imaging. After undergoing the standard histopathological specimen processing and imaging procedures, we obtained Hematoxylin and Eosin (H&E) images for both TSCC and normal tissues. The duration for this processing stage was ~14 h. **d** Consistency assessment was conducted by three pathologists who independently reviewed the generated H&E images and provided classifications for the corresponding tissue samples. By comparing these classifications with those obtained from real H&E images, we were able to evaluate the consistency of histopathological conclusions drawn using the generated H&E images

In this study, we concurrently collected tissue samples from the tongues of 15 patients with TSCC and the normal tissues from the bottom of the excised tumors during surgery (Fig. 1a). Both tissue types underwent histopathology imaging and Raman imaging, yielding corresponding H&E images and Raman images. These images were utilized to establish a generative AI model for transforming from Raman images to H&E images. Tissue samples were directly placed on the Raman imaging instrument for scanning after cryosectioning. To ensure comprehensive coverage of the scanning area, we selected eight scanning regions, each with dimensions of 50 by 400 µm, for every tissue slide. The scanning time for each region was 2.5 min, resulting in the acquisition of a Raman image upon completion (Fig. 1b). Meanwhile, the collected TSCC tissues and surrounding normal tissues also underwent the standard histopathology specimen process, encompassing fixation, dehydration, embedding, sectioning, and staining. Subsequently, the tissues underwent histopathology imaging, generating H&E images for both TSCC and normal tissues. The entire process took ~14 h (Fig. 1c). Comparative analysis of sample processing complexity revealed a significant simplification in the sample processing workflow for Raman imaging. Unlike the time-consuming and intricate histopathology specimen process required for histopathology imaging, the Raman imaging time for a single scanning area (50 μm × 400 μm) has been reduced from hours to minutes, significantly decreasing the time needed to generate digital images for the tissue.

### Histopathological characteristics of H&E images and Raman images

Supplementary Fig. 1a clearly illustrates the H&E images of TSCC tissue and normal tissue obtained through histopathology imaging. In the H&E image of TSCC tissue (left panel), distinctive histopathological features crucial for TSCC diagnosis are clearly visible,^38,39^ which include: (1) Pleomorphism of squamous cells, characterized by variability in size, shape, and staining. The nuclei may appear enlarged and irregular, with more prominent nucleoli. (2) Formation of keratin pearls, indicative of cellular keratinization. Keratin pearls are concentric circular structures formed by mature squamous cells. (3) Invasion of cancer cells from the primary site into surrounding connective tissue, a key feature of TSCC’s aggressive growth, corresponding to marked regions #1, #2, and #3, respectively. In the H&E image of normal tissue (right panel), characteristic histopathological features of normal tongue muscle tissue are also easily identifiable. These features include: (1) The muscle cells of the tongue exhibit a typical striated (striped) structure. (2) Similar to other skeletal muscles, tongue muscle cells are elongated and typically contain multiple nuclei located at the periphery of the cell; (3) The tongue muscle tissue also contains connective tissue, which helps support and connect muscle fibers and provides pathways for nerves and blood vessels, corresponding to marked regions #1, #2, and #3, respectively. By comparing the histological features displayed in the H&E images of TSCC tissue and normal tissue, pathologists can effectively distinguish malignant tissue from normal tissue.

Raman imaging can visually represent the relative abundance of various substances in soft tissues through the grayscale of images. Supplementary Fig. 1b presents Raman images scanned in eight regions of TSCC tissue (left panel) and normal tissue (right panel). We utilized the full spectral range (600–3 100 cm^−1^) for visualization, and to facilitate observation, the images were processed to eliminate interference from tissue autofluorescence. The images reflect the compositional variations of typical substances in tongue tissue, such as proteins, lipids, fatty acids, and other organic matters, in different scanning regions. They also outline the general profiles of muscle cells in normal tissue. However, when comparing Raman images of TSCC tissue and normal tissue, the changes in substance composition reflected may not allow pathologists to intuitively observe the histopathological characteristics of TSCC or assess tissue lesions, even if there are certain differences between the two images of tissue types. Therefore, despite the advantages of fast and non-destructive scanning, Raman images are challenging to be directly used for pathological diagnosis in clinical settings. They need to be visualized to depict the histopathological conditions of the scanned tissues, such as being converted into H&E images.

The generated H&E images were independently reviewed and classified by three pathologists. To assess diagnostic accuracy, conclusions drawn from real H&E images of the tissues were used as the ground truth and compared with those drawn from the generated H&E images. (Fig. 1d).

### AI generation model development

To generate H&E images, we developed a CLIP-like model, named ImgAlignNet, bridges Raman images with H&E images to provide conditioning information, which then directs a diffusion model in generating the H&E images. The overview of procedures of the model development and H&E image synthesis are shown in Supplementary Fig. 2. The ImgAlignNet uses the mechanism of ViT to segments the images into patches and uses the concept of vector quantized-variational auto-encoder (VQ-VAE) and support vector machine (SVM) for feature capturing and classification under a latent space by using trainable targets. The training process was divided into two steps, initially the ImgAlignNet was trained as a multi-task classification model for both H&E and Raman images, then the optimized targets was used as conditioning for training the diffusion model. The algorisms of ImgAlignNet and diffusion model were introduced in “Method” section, and the details of implementation were presented as pseudo code in Supplementary Methods. After training, the captured feature regions can be visually highlighted in the original images, and the conditioning information can be generated unsupervised based on an attention-like operation.

We utilized samples obtained from twelve early-registered patients, which included H&E images and Raman images. The dataset consists of a total of 24 H&E images, with 12 images representing TSCC tissues and 12 images representing normal tissues, accompanied by 192 corresponding Raman images. This dataset served as the training and validation set for the development of an AI transformation model. Subsequently, the remaining data from three later-registered patients, comprising both H&E images and Raman images, were utilized as an independent test set to assess the model’s capability in generating H&E images. This test set comprises six H&E images each of TSCC tissue and normal tissue, along with 48 corresponding Raman images.

### Generation outcomes using Raman images from different tissue regions

**Fig. 2:**
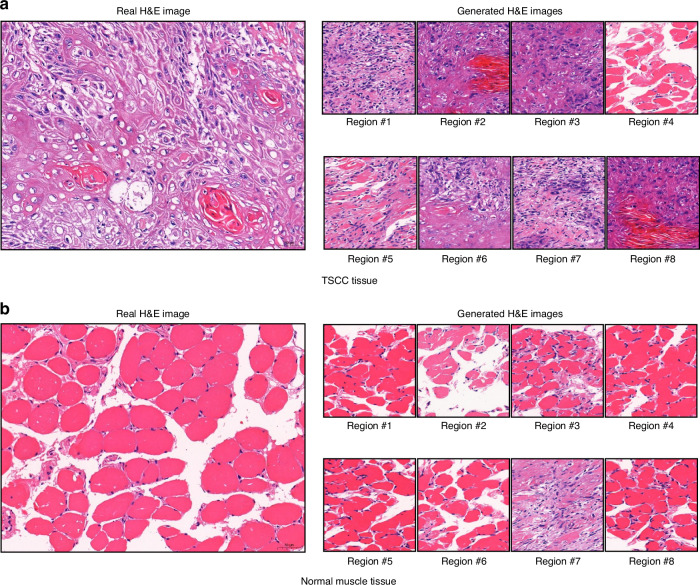
Comparison between real H&E images and generated H&E images of TSCC and normal muscle tissue. **a** Presents the real H&E image alongside generated H&E images depicting eight regions of TSCC. **b** Presents the real H&E image in comparison with generated H&E images depicting eight regions of normal muscle tissue

To ensure comprehensive coverage of Raman imaging across both TSCC and normal tissues, we carefully selected eight regions on each tissue for scanning. Figure 2 presents a set of eight H&E images for TSCC tissues and eight H&E images for normal tissues. In the case of TSCC tissue (Fig. 2a), the generated H&E images of seven regions are similar to the real H&E image. The generated H&E image of region #4 is more like normal muscle tissue. Upon examining our model’s structure (Fig. 2), the process of H&E image generation entails mapping the input Raman image to pretrained Raman image targets, aligning the mapping results to corresponding H&E image targets, and utilizing the linearly combined/synthetic H&E image targets as conditioning for H&E image generation. Bias may manifest during mapping to pretrained Raman image targets when the Raman signals in the scanned area lack distinctive histological features, leading to inaccuracies in H&E image generation. Similar observations apply to the results obtained for normal muscle tissue (Fig. 2b). Note that our model utilizes a diffusion-based framework to generate the final H&E image. Each generated H&E image may exhibit slight variations, resulting in diverse outcomes (Supplementary Fig. 3).

### Diagnostic outcomes utilizing generated H&E images

**Fig. 3:**
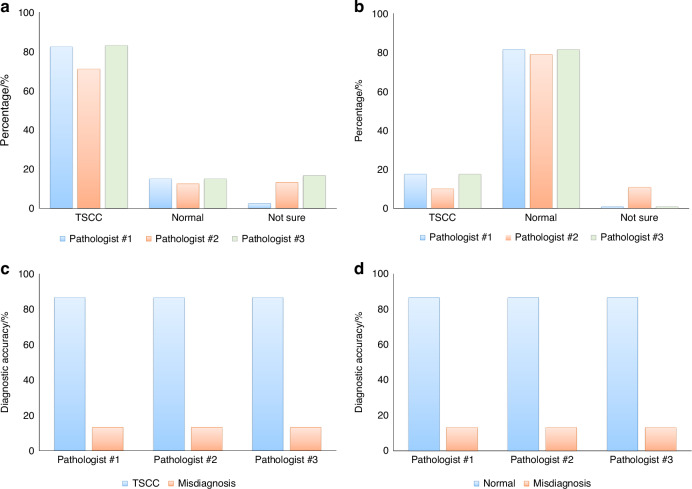
The consistency in tissue classification between real and generated H&E images. Using real H&E images as the ground truth, three pathologists independently reviewed the generated H&E images, determined tissue categories, and compared their assessments with the ground truth to calculate diagnostic accuracy. **a** Results of tissue category assessments for 120 regions of generated H&E images from 15 TSCC samples. **b** Results of tissue category assessments for 120 regions of generated H&E images from 15 normal muscle tissue samples. **c** Diagnostic accuracy of TSCC determined by the three pathologists using generated H&E images. **d** Diagnostic accuracy of TSCC determined by the three pathologists using real H&E images as the ground truth

The three pathologists individually reviewed the generated H&E images and provided conclusions regarding tissue classification, categorizing them as TSCC, normal muscle tissue, or expressing uncertainty. Results were collected from the evaluations of 240 regions across 30 samples by the pathologists. Figure 3a, b depict the consistency between the pathologists’ assessments of TSCC and normal muscle tissue in different regions and the corresponding real H&E images. The figures illustrate an approximately 80% concordance between the pathologists’ assessments of the generated H&E images and the real H&E images. Based on the evaluations of eight regions, if more than four regions align with the real H&E images for a given sample, we consider the diagnosis correct; otherwise, it is considered as misdiagnosis. Figure 3c, d illustrate the diagnostic accuracies of the three pathologists for 15 TSCC samples and 15 normal muscle tissue samples, both reaching 86.7%.

**Fig. 4:**
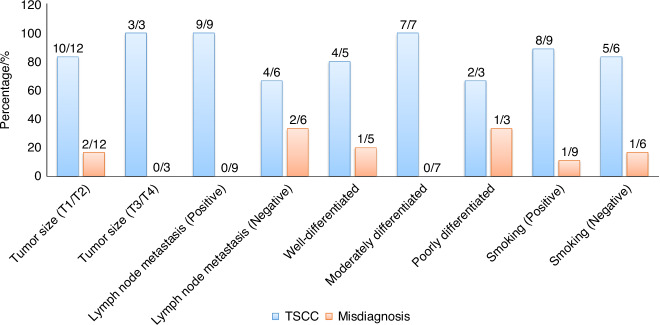
Accuracy and misdiagnosis rates of generated H&E images in the assessment of TSCC samples under specific clinical indicators

Furthermore, our model demonstrates variations in the discriminative ability of the generated H&E images for tumor samples under different clinical indicators. We conducted an analysis using univariate analysis of variance to evaluate the results (Supplementary Fig. 4). Notably, TSCC samples with specific clinical indicators such as lymph node metastasis or histologic grade exhibited significant variations in the generated H&E images (Fig. 4). Considering clinical indicators like large tumor size, positive lymph node metastasis, and moderately differentiated grade, pathologists achieved a 100% diagnostic accuracy upon reviewing these images (Fig. 3).

### Generated results at specific stages of tumor progression

**Fig. 5:**
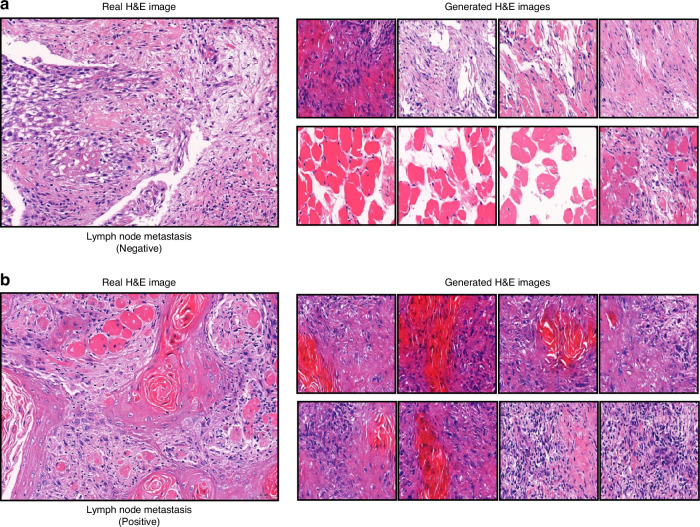
Real and generated H&E images of TSCC samples by lymph node metastasis. **a** Real and generated H&E images of TSCC samples by negative lymph node metastasis. **b** Real and generated H&E images of TSCC samples by positive lymph node metastasis

We conducted a further investigation into the model’s ability to generate H&E images for TSCC samples with varying lymph node metastasis statuses (positive vs. negative) and different histologic grades. Fig. 5a, b present the results for TSCC samples with negative and positive lymph node metastasis, respectively. It is apparent that for the TSCC sample with negative lymph node metastasis, the real H&E images primarily showcase the pleomorphism of squamous cells and the invasion of cancer cells into connective tissue. The model exhibits suboptimal performance in generating H&E images for such sample. In contrast, for the TSCC sample with positive lymph node metastasis, the images not only exhibit characteristics observed in samples with negative lymph node metastasis but also distinct features such as the formation of keratin pearls. Our model effectively captures these features, resulting in more accurate generated H&E images.

**Fig. 6:**
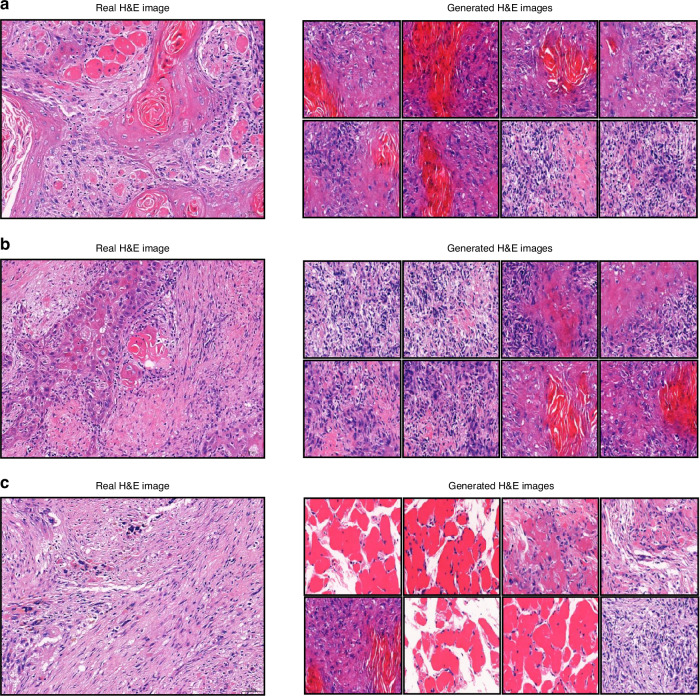
Real and generated H&E images of TSCC samples stratified by histologic grades. **a** Well-differentiated grade. **b** Moderately differentiated grade. **c** Poorly differentiated grade

Figure 6a–c illustrate the model’s generation of H&E images for TSCC samples with well-differentiated grade, moderately differentiated grade, and poorly differentiated grade, respectively. The figures reveal significant changes in the primary features of the TSCC samples as they transition from well-differentiated to poorly differentiated grade, including notable variations in the morphology of keratin pearls and squamous cells. For well-differentiated and moderately differentiated grade TSCC samples, the model accurately generates corresponding histopathological features. However, for poorly differentiated grade TSCC sample, the model tends to produce inaccurate images, with four regions out of eight generating an H&E image that more closely resembles normal muscle tissue.

### Spatial characteristics represented by H&E targets

The pivotal role of H&E targets as conditioning factors in guiding the diffusion model for H&E image generation is paramount. Using sample #1 from the independent test set as an illustrative case, we delve deeper into the spatial features embodied by H&E targets in real H&E images. Pretrained H&E targets are paired with H&E patches, obtained through segmentation of the real H&E image, to form target-patch pairs. The cosine similarity between the target and patch in each pair is calculated, and all target-patch pairs are ranked based on their similarity scores in descending order. Subsequently, the top *n* patches are selected and overlaid onto the real H&E image. This approach enables us to discern the spatial features captured by H&E targets and employed in the generation of the final H&E image. Supplementary Fig. 5 provides a visual representation of the corresponding results on spatial features for TSCC and normal tissues, achieved by selecting the top 256, 512, 1 024, and 2 048 patches, respectively. Analyzing the results for TSCC tissue (Supplementary Fig. 5a) reveals that the represented features commence with invaded connective tissue by tumor cells and squamous cells exhibiting various sizes, shapes, and staining. As the number of patches increases, these features progressively cover the majority of areas in the image, incorporating intricate details such as keratin pearls. For normal tissue (Supplementary Fig. 5b), the represented features initiate with connective tissue between normal muscle cells, gradually extending to cover the nuclei located at the periphery of the cell and finally intricately detailing the striated structure of muscle cells. This outcome indicates that our model, in its training process, strategically prioritizes learning the morphological disparities in connective tissue between TSCC and normal tissues. Subsequently, it adeptly captures the morphology and variations in the structure of muscle cells, along with TSCC-specific features.

## Discussion

Raman spectroscopy, compared to conventional histopathology for disease diagnosis, can detect changes in the molecular structure or composition of biomolecules within cells even before significant morphological alterations occur. Hence, it is referred to as a “molecular fingerprint” and is increasingly being applied in the differential diagnosis of diseases.^40–42^ Currently, Raman spectroscopic studies on diseased tissues primarily focus on the comparison and analysis of linear spectra. Although this method is rapid and straightforward, it only investigates a few points of interest within the diseased tissue area rather than the entire area, which may not accurately reflect the Raman spectral characteristics of the diseased tissue. Raman mapping, representing the characteristics of a specific area, offers a more representative approach and a greater volume of spectral data.

This approach allows for the transformation of Raman spectroscopic images into visual histopathological images, eliminating the need for conventional tissue section preparation and staining processes. This not only saves time but also avoids contamination from biological reagents,^43^ presenting tumor histopathological characteristics more rapidly and accurately to clinical pathologists. Furthermore, with ongoing research and the continuous refinement of computational models, this approach can increasingly assist pathologists in diagnosing tumors and even in differentiating between tumor pathological subtypes.

Furthermore, the proposed approach successfully differentiates between tongue squamous carcinoma tissues and normal muscle tissues, which holds significant clinical importance for the assessment of surgical margins in tongue squamous cell carcinoma. It is well-known that the status of surgical margins in tongue squamous cell carcinoma has a profound impact on patient prognosis, with positive margins often leading to local recurrence. The evaluation of deep surgical muscle margins in tongue squamous cell carcinoma presents a challenge; on one hand, achieving negative surgical margins necessitates the removal of a sufficient amount of muscle tissue, while on the other hand, to preserve function, as much muscle tissue as possible should be conserved.^5,10^ Therefore, this technology has immense clinical application potential in assessing deep surgical margins in tongue squamous cell carcinoma.

In addition, ImgAlignNet, developed to capture feature regions for classification in a dataset with a limited number of samples, exhibits a preference for color-based features. In TSCC images, the model primarily focuses on regions with variable staining of squamous cells and connective tissue invasion, characterized by a mix of purple and red hues. Conversely, in normal images, it tends to highlight intercellular spaces and normal connective tissue, which often appear white and light red. This color-focused approach may inadvertently downplay the importance of keratin pearls, predominantly white and light red, colors typically associated with normal image regions, even though they are commonly key features of TSCC.

As demonstrated in the results section, our diffusion model, like others using attention mechanisms, generates distinct H&E images for each iteration, even with identical conditions. This variability arises from the model’s learned associations with various sub-regions during training. Since these associations are not uniquely fixed for a given region, the resulting generation varies. This variability in generation can be viewed as both a feature and a limitation, depending on the specific application of the generated images.

To enhance the model’s ability to capture a broader range of feature regions and ensure more consistency in the images generated from the same conditioning information, an increase in the number of samples and more detailed pixel-level labeling are required. Such labeling will not only facilitate the capture of predominant features like squamous cells, connective tissue, and intercellular spaces, but also enable the detection and generation of muscle cells, keratin pearl regions, and even image boundaries. This can be achieved through location-specific conditioning in the reverse loop. Additionally, reverting ImgAlignNet to a transformer-based model could further improve its capability to capture and process conditioning information effectively.

While the current dataset has proven sufficient for the generation of H&E images distinguishing between tumor and normal tissues, particularly given the well-defined pathological features of tongue cancer, it is important to acknowledge that more detailed classification tasks, such as identifying the differentiation grades of tongue cancer, would likely require a larger sample size. A greater number of patient samples would help capture the finer histological variations that are necessary for accurately distinguishing between high, moderate, and poorly differentiated cancer tissues. Increasing the dataset would enable the model to generate more precise and nuanced features, ultimately enhancing its performance in more complex diagnostic scenarios. Thus, future studies will specifically focus on expanding the sample size to further improve the model’s ability to generate high-resolution, clinically relevant details for these advanced classification tasks.

## Materials and methods

### Patient cohort and demographics

This study involved a cohort of 15 patients diagnosed with TSCC who were admitted to our facility between January and December 2023. All participants had received pathological confirmation of TSCC before their inclusion in this study. None of the enrolled individuals exhibited any other systemic diseases or a history of medication use. Prior to the commencement of the study, informed consent was obtained from all patients, and the research protocol received approval from the Ethics Committee of the First Affiliated Hospital of Xiamen University (Grant No. XMYY-2022KYSB030) and the Ethics Committee of West China Hospital of Stomatology, Sichuan University (Grant No.WCHSIRB-CT-2023-438). Among the 15 TSCC patients, 12 were admitted in the early stage of 2023 (from January to July of 2023) and were subsequently assigned to our training-validating process. The remaining three patients, newly diagnosed and admitted in late 2023, were allocated to the external test set for model evaluation. Demographic information for the TSCC patients is detailed in Supplementary Table 1.

### Preparation of tissue slides

Tissue samples from patients with TSCC and corresponding normal muscle tissues were obtained from surgical resection specimens in the operating room. The normal muscle tissues were specifically harvested from areas located 5 mm beyond the tumor margins. Both the tumor and muscle tissues underwent processing using the freezing microtome to generate standard sections, subsequently subjected to H&E staining for histopathological confirmation. Simultaneously, 6-µm-thick tissue sections from the same specimens were sliced using the freezing microtome and affixed to custom-made pure aluminum slides for subsequent Raman spectroscopic scanning. In total, 30 H&E-stained slides were prepared for histopathological imaging and confirmation, along with an additional 30 slides for Raman imaging, derived from the samples of 15 patients diagnosed with TSCC.

### Generation of H&E images

The fixed tissue samples underwent a systematic dehydration process utilizing an automatic dehydrator. The dehydration sequence involved sequential immersion in various alcohol concentrations, totaling 5 h and 20 min: 75% alcohol for 1 h, 85% alcohol for 1 h, 95% alcohol I for 50 min, 95% alcohol II for 50 min, 100% alcohol I for 50 min, and 100% alcohol II for 50 min. Subsequently, a mixture comprising 100% alcohol and xylene in a 1:1 ratio was applied for 20 min, followed by xylene I for 25 min and xylene II for 25 min. To achieve optimal embedding, the tissues underwent additional steps, including immersion in paraffin I for 1 h, paraffin II for 2 h, and paraffin III for 3 h. Following this comprehensive dehydration and embedding process, the tissues were prepared for sectioning.

For deparaffinization, sections underwent a meticulous process involving xylene treatment. This included immersion in xylene I for 5–10 min, followed by xylene II for 5–10 min. Subsequently, the sections underwent sequential immersions in absolute ethanol I for 5 min, absolute ethanol II for 5 min, 95% alcohol for 5 min, 85% alcohol for 5 min, and 75% alcohol for 5 min. Finally, the sections were soaked in UP water for 5 min, completing the deparaffinization process. This systematic approach ensures the removal of paraffin, preparing the sections for further analysis.

Following deparaffinization, the sections underwent a staining process to highlight cellular structures. Initially, the sections were stained with hematoxylin for 10–20 min, then rinsed with tap water for 1–3 min. Subsequent differentiation occurred in hydrochloric acid alcohol for 5–10 s, followed by another rinse with tap water for 1–3 min. The sections were then blued in warm water at 50 °C or a weak alkaline aqueous solution until achieving a blue coloration. After a thorough tap water rinse for 1–3 min, the sections were immersed in 85% alcohol for 3–5 min. Eosin staining followed, lasting 3–5 min, and another rinse with tap water for 3–5 s. The subsequent steps included dehydration in graded alcohols, clearing in xylene, and finally, mounting with neutral resin.

### Raman imaging for tissue sections

Raman imaging of tissue slides was conducted using the Nanophoton Raman-11 laser Raman microscope (Nanophoton, Japan). The slides were precisely positioned on the motorized XYZ stage to ensure precise spatial alignment. To achieve comprehensive coverage, eight regions of interest were systematically recorded across various sections of the slides. The 532 nm excitation laser beam was focused onto the sample using an X20 0.45 NA Nikon lens. Linear scanning imaging was employed to generate Raman images of the tissue samples, with scanning parameters meticulously configured as follows: a lateral range of 400 µm and a vertical range of 50 µm, both with a resolution of 1 µm. Lateral linear scanning was executed with an exposure time of 3 s, maintaining a power level of ~0.2 mW. Each region underwent scanning for a duration of 2.5 min.

### ImgAlignNet model

As a key component of our methodology, the ImgAlignNet (Supplementary Fig. 2b), analogous to CLIP, captures and aligns features from Raman and H&E images. The ImgAlignNet was developed to address two challenges:

1. Different from text-image model, which generally relies on spatial information from only image modal, in this study, the ImgAlignNet needs to manage the inherent spatial information in both Raman images and H&E images, focusing on aligning their local contents.
2. Limited data in clinical settings constrains the ability of Transformer architectures which require extensive parameter tuning and benefit from training on larger datasets. Thus, the ImgAlignNet should have the ability to handle small datasets.

To address the alignment challenge, the model adopts ViT’s segmentation strategy to partition the encoded spatial data into patches. It then leverages a unique approach, inspired by VQ-VAE, where the same seed is used to generate corresponding targets in both Raman and H&E latent spaces, ensuring coherent alignment. Since the targets can be aligned by using the same seed, the CLIP’s contrastive loss can be replaced by a basic binary classification loss for TSCC/normal classification to avoid the issue of limited samples. To further address the challenge of limited sample size, an SVM-inspired classification framework, utilizing the cosine similarity as distance measurement between patches and their corresponding targets, was incorporated into the model.

In more detail, data from both modalities are first downsampled using a CNN-pooling architecture, reducing the dimensionality, and thus easing the computational load for subsequent processes. The data and images are then segmented into patches using the ViT strategy, resulting in ${Patc}{h}_{R{A}_{i}}$ for Raman images and ${Patc}{h}_{H{E}_{j}}$ for H&E images, where $i$ and $j$ represent the indices of the respective patches. After segmentation, each patch is flattened into a one-dimensional vector with the dimensionality of $d$, where $d$ equals the product of patch size ${sH}\times {sW}$ and the number of channels $c$ after the last convolution layer. Consequently, the seeds are initialized to a dimension ${d}_{{seed}}$, and targets of the dimension $d$ to match the patches can be generated using a few fully connected layers with nonlinearity operations from the seeds. Specifically, the seeds are associated with the final classes (i.e., normal and cancer), leading to the generation of targets within their respective latent spaces:

1 $${Targe}{t}_{{cls},k}^{M}={FC}{s}^{M}\left({see}{d}_{{cls},k}\right)$$

Where ${cls}\in \left\{{TSCC},{normal}\right\}$, $M\in \left\{{Raman\; image},\mathrm{H\& E\; image}\right\}$ and $k$ is the index of seed and targets. With the targets established, distances in each class can be calculated as follows:

2 $${dis}{t}_{{cls},i,k}^{M}=\cos {Similarity}\left({Patc}{h}_{{M}_{i}},{Targe}{t}_{{M}_{{cls},k}}\right)$$

Where and $i$ serves as a general index for patches in both modalities, corresponding to index $i$ for ${Raman\; image}$ and $j$ for $\mathrm{H\& E\; image}$ in the original data. Based on the distances, the logits can be calculated using the equation:

3 $$logit{s}_{cls}^{M}=\sum _{k}{{\rm{w}}}_{cls,k}^{{\rm{M}}}\sum _{i}\exp (\lambda (1+dis{t}_{cls,i,k}^{M}))$$

Where ${{\rm{w}}}_{{cls},i}^{{\rm{M}}}$ and $\lambda$ are trainable nonnegative parameters, denoting the weight and temperature factor, respectively. Equation $\left(3\right)$ initially scales the range of cosine similarity values from $\left[-\mathrm{1,1}\right]$ to $\left[\mathrm{0,2}\lambda \right]$. The exponential function applied after scaling predominantly amplifies larger values, making the large values more influential across feature axis. Following this, a weighted summation across the target axis is performed to compute the logits. The final loss value is calculated as follows:

4 $${los}{s}^{M}={crossEntropy}\left({softma}{x}_{{cls}}\left({logit}{s}_{{cls}}^{M}\right),{labe}{l}^{M}\right)$$

The equations above ensure that in the model’s assessment, the focus is more on the larger cosine similarity values, reflecting the importance of the large similarity between patches and targets in the overall calculation.

### Diffusion model

The diffusion model, another key component in our framework, is employed to generate H&E images (Fig. 2b). The diffusion model is trained using noised images and corresponding conditioning information, with the objective of transforming a random noise distribution into a coherent image structure during the inference process. In our approach, the primary effort involves integrating the ImgAlignNet model’s outputs into this diffusion process, which enhances the model’s ability to generate H&E images in a more controlled and less random manner. This enhancement is achieved by utilizing the features extracted from Raman images differently in the training and inference stages.

In more detail, during the training phase of the diffusion model, each diffused H&E image is combined with all corresponding ${Targe}{t}_{{cls}}^{{HE}}$ that share the same class label, serving as conditioning inputs. While in the inference phase, the conditioning is generated from a Raman image. Initially, the distances ${dis}{t}_{{cls},i,k}^{{RA}}$ are computed according to equation$\left(3\right)$, where $i$ and $k$ are the indices of patches and targets, respectively. Then, a masking mechanism is applied to filter out smaller distance values using the equation:

5 $$\begin{array}{l}mas{k}_{cls,i,k}^{RA}=\left\{\begin{array}{l}\,1\,if\,dis{t}_{cls,i,k}^{RA}\, > \,thre{s}_{cls,i,k}\\\ \!-inf\,otherwise\,\end{array}\right.\end{array}$$

In equation $\left(5\right)$, ${thre}{s}_{{cls},i,k}$ can vary across the three axes, indicating a dynamic threshold. After experimenting with various approaches, a two-step top-K selection process was determined for setting the thresholds. This process initially selects the kth largest values along the patch axis, and subsequently performs another top-K selection along the combined class and target axis. Having obtained the mask, the conditioning for the inference process is computed in conjunction with the aligned H&E targets using the equation:

6 $$con{d}_{k}=\,\sum _{cls,i}softma{x}_{cls,i}(mas{k}_{cls,i,k}^{RA}\cdot dis{t}_{cls,i,k}^{RA})Targe{t}_{cls,k}^{HE}$$

Here, ${softma}{x}_{{cls},i}$ denotes the SoftMax operation applied along the combined axis of class and targets. Equations $\left(5\right)$ and $\left(6\right)$ are based on the attention mechanism, where the attention matrix is computed using a temperature-modified cosine similarity distance. Additionally, a sparse filter is applied to eliminate smaller values. After filtering to retain only the larger distance values, a *SoftMax* operation is applied to these values, emphasizing the most significant interactions. This refined data is then linearly combined with the aligned H&E targets to guide the image generation in the reverse diffusion loops.

### Model implementations

In total, for training ImgAlignNet, 88 × 20 = 1 760 H&E sub-images and 8 × 20 = 160 Raman sub-images were generated for the training dataset; 88 × 4 = 352 H&E sub-images and 8 × 4 = 32 Raman sub-images were generated for the validation dataset; and 88 × 6 = 528 H&E sub-images and 8 × 6 = 48 Raman sub-images were generated for the testing dataset. For the diffusion model, the number of generated H&E sub-images increased to 180 × 20 = 3 600 for the training dataset, 180 × 4 = 720 for the validation dataset, and 180 × 6 = 1 080 for the testing dataset. The number of generated Raman sub-images remained the same as those used for training ImgAlignNet.

The detailed aspects of our model development are depicted in Fig. 2b, and the pseudo code was provided in the Supplementary Methods. Firstly, the H&E images with shape $\left[{3,1\;304,980}\right]$ were processed to the same resolution where 1 pixel equal to $1\mu {m}^{2}$ to be the same as Raman images with shape $\left[{1\;340,400,50}\right]$ by using the resize operation from torchvision library. The shape of H&E images becomes $\left[\mathrm{3,602,490}\right]$ after the resizing operation. Then an unfold-like operation was developed to extract sub-images from the processed images. For the ImgAlignNet model, the H&E sub-image has the shape of $\left[\mathrm{3,256,256}\right]$ with a stride of 32 pixels in both height and width, and the Raman sub-image has the shape of $\left[{1\;340,400,48}\right]$ without stride. For the diffusion model, the shape of H&E images becomes to $\left[\mathrm{3,128,128}\right]$ and others keep to the same.

Secondly, the ImgAlignNet model employs separate initial convolution layers for processing Raman and H&E images, respectively. The outputs from these layers are then individually processed through respective CNN downsampling blocks. Each downsampling block is composed of two residual CNN blocks, followed by a downsampling layer. The residual CNN block comprises two convolution layers, each preceded by a groupNorm layer followed by a SiLU activation layer. Across each residual CNN block, a residual shortcut is implemented by summing the block’s input with the output from its last convolution layer. The final downsampling layer is achieved using a CNN layer with a stride of 2. Using the two downsampling blocks, the dimensions of the H&E image were reduced from $\left[3,\,256,\,256\right]$ to $\left[64,\,64,\,64\right]$, and those of the Raman images from $[1\;340,\,400,\,48]$ to $[64,\,100,\,12]$. Subsequently, ViT segmentation was employed to split the data into 256 and 75 patches of size $[64,\,4,\,4]$ for H&E and Raman images, respectively. Seed arrays were generated from independent normal distributions with a dimension of 64, and then expanded to a dimension of 128 through three fully connected layers. Between each pair of fully connected layers, a layerNorm layer and a SiLU layer were inserted. The patches were then flattened and projected to a size of 128 by another fully connected layer for the cosine similarity calculation and subsequent classification. The temperature factor λ was initialized at $\mathrm{log}\left(2\right)\approx 0.693\;1$, and a label smoothing mechanism with a value of 0.15 was employed for the final cross-entropy computation. Specifically, after exploring various approaches, we decided to pre-train the CNN downsampling blocks for H&E images using an auto-encoder. This auto-encoder is composed of CNN downsampling blocks, each paired with a corresponding upsampling block, differing only in the final layer where the downsampling layer is replaced by upsampling layer. When training the ImgAlignNet model, we extracted and froze the downsampling blocks from this pretrained auto-encoder to abstract the image features. This freezing was found to be crucial, as it prevented overfitting, which was evident from observing the validation dataset loss values. Contrarily, using a pretrained auto-encoder for Raman images, based on our tests, was found to adversely affect classification performance, even reducing it.

Finally, the diffusion model employs a UNET structure to synthesize the H&E image with the Raman targets as conditioning. It comprises five CNN downsampling blocks, each reducing the image dimensions to half, and an equal number of upsampling blocks, sequentially restoring the image back to its original size. Following the common UNET design, outputs from the downsampling blocks are also utilized as shortcut inputs for the corresponding up-sampling blocks by concatenating these outputs with the respective inputs of the up-sampling pathway. Specifically, self-attention and cross-attention layers, placed before the down/up-sampling layers, are selectively implemented when the H&E image resolution is reduced to 16 × 16 or smaller, in consideration of balancing the computational burden. When training the diffusion UNET, the Raman targets are integrated as conditioning through these cross-attention layers.

### The running environment and parameters

During training, the *AdamW* optimizer was used for both models with an initial learning rate of 1E-5 and a decay rate of 1E-5. MSE (mean squared error) loss was employed for both the auto-encoder and diffusion models, while cross-entropy loss was used for the ImgAlignNet model. Since the ImgAlignNet model is designed as a multi-task classification model, the accuracy score was used to evaluate the classification performance. Training was conducted on up to 16 NVIDIA 3080 GPUs in a cluster, each with 10 GB of graphics memory. All models were developed using PyTorch, with version 1.13 utilized in the cluster environment and version 2.0 for local development. The frequency of backward operation was set to occur every 64 samples, equivalent to a local mini-batch size of 64, achieved by utilizing the distributed data parallel (DDP) module and gradient accumulation, regardless of the varying number of GPUs. For the ImgAlignNet model, considering the limited dataset size, we implemented an early stopping mechanism with a threshold of 300 epochs (about 10 000 steps), monitored by the minimal loss value on the validation set. In contrast, the diffusion model was trained for a total of 1 000 epochs (equivalent to 33 000 steps), with checkpoints saved every 25 epochs. The best validation set checkpoint of the diffusion model was preserved as well, even though early stopping was not employed.

## Supplementary information

Supplemental material

## Data Availability

This study protocol was approved by the ethics committee of the first affiliated hospital of Xiamen University. Since the human genetic resources management requirements of the institution, we only publicly provide the H&E and Raman images of one anonymized patient. For reasonable data security concerns, the data is only available to research collaborators approved by the Ethics Committee of the first affiliated hospital of Xiamen University and the Ethics Committee of West China Hospital of Stomatology, Sichuan University. Researchers interested in participating in the collaboration should contact the corresponding authors for approval. The data, together with the related code are available at: https://github.com/jingry/HE-RA-alignment-and-generation.
