# Supplementary material for: GenAI synthesis of histopathological images from Raman imaging for intraoperative tongue squamous cell carcinoma assessment
Source: Int J Oral Sci. 2025 Jan 26;17:12. doi: 10.1038/s41368-025-00346-y (PMC11770123; doi:10.1038/s41368-025-00346-y)
Supplement: Supplementary file 1 — Supplemental material [file 41368_2025_346_MOESM1_ESM.pdf]

Supplementary Figures

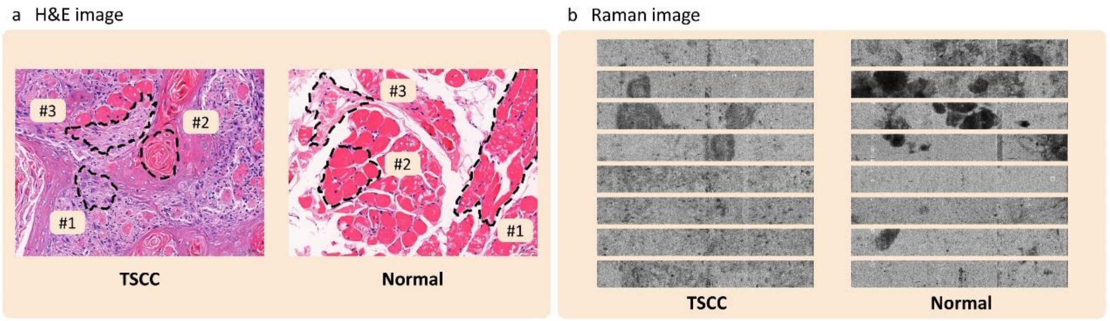

Supplementary Fig 1: H&E images and Raman images generated by histopathological imaging and Raman imaging, respectively. a Histopathological characteristics in H&E images. Left Panel: Significant histopathological features of TSCC observed in H&E images include pleomorphism of squamous cells (#1), formation of keratin pearls (#2), and invasion of cancer cells into surrounding connective tissue (#3). Right Panel: H&E images of normal tissue reveal major characteristics such as tongue muscle cells with a striated (striped) structure (#1), multiple nuclei positioned at the periphery of the cell (#2), and connective tissue (#3). b Raman images of TSCC and normal tissues. It posed a challenge to discern clear histopathological characteristics from these images.

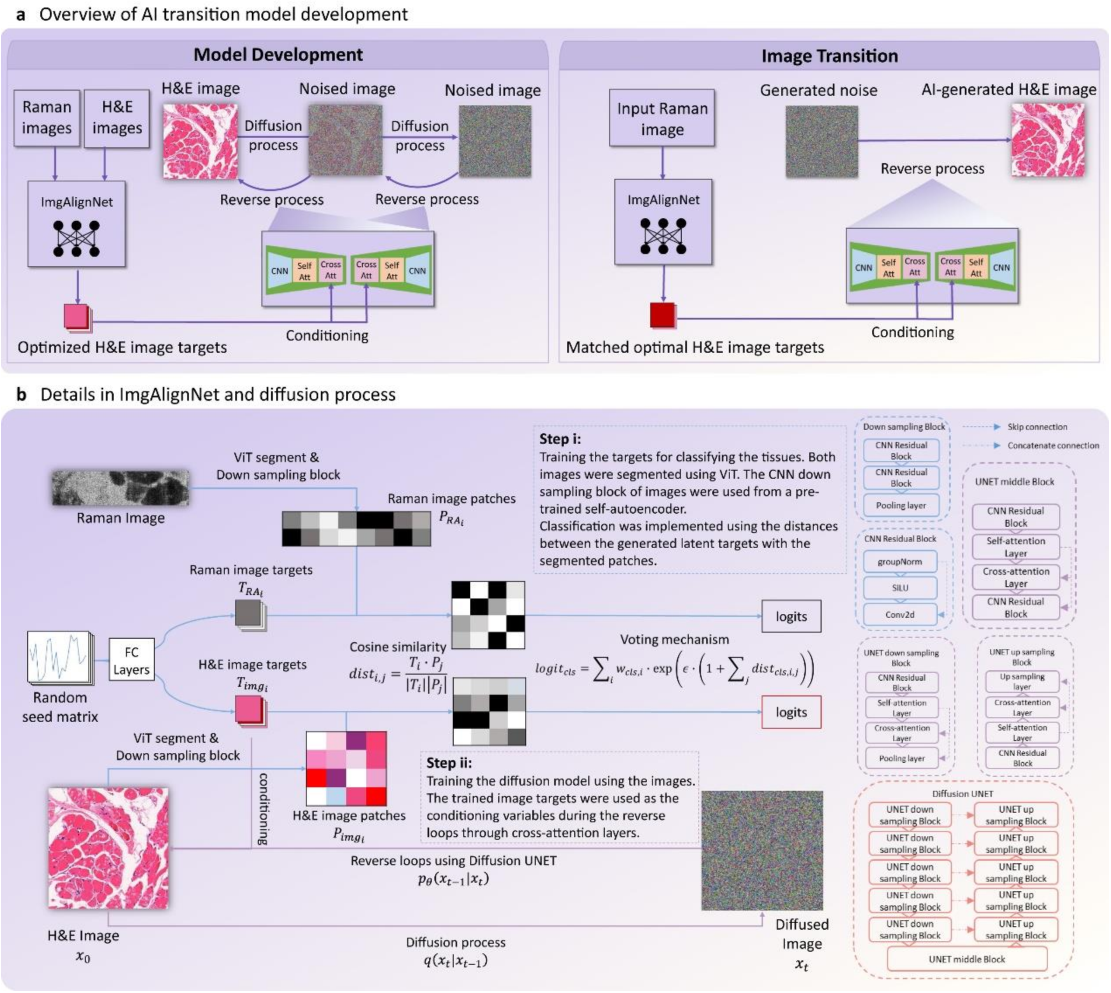

Supplementary Fig 2: Overview and Detailed Processes of AI Transformation Model. a Overview of the AI transformation model. In the model development phase (left panel), ImgAlignNet underwent training utilizing both Raman and H&E images. Specifically, ImgAlignNet was trained to align Raman images with H&E images. The H&E targets was pre-trained (optimized) for conditioning in the reverse process of the diffusion model training. In the subsequent application of the model for image transformation (right panel), the input Raman image underwent alignment with the pre-trained (optimized) H&E targets. These optimized targets were then utilized as conditioning to guide the diffusion model in generating an H&E image. b Details in ImgAlignNet and diffusion processes. Within the detailed processes, both Raman and H&E images were separately segmented and down-sampled into patches. Fully Connected (FC) layers were trained to generate optimized targets. These targets were strategically designed to exhibit the largest cosine similarity with corresponding patches and the highest accuracy in classifying TSCC and normal tissues. Subsequently,

the pre-trained (optimized) targets, known as H&E targets, played a crucial role as conditioning in the reverse process of developing the diffusion model.

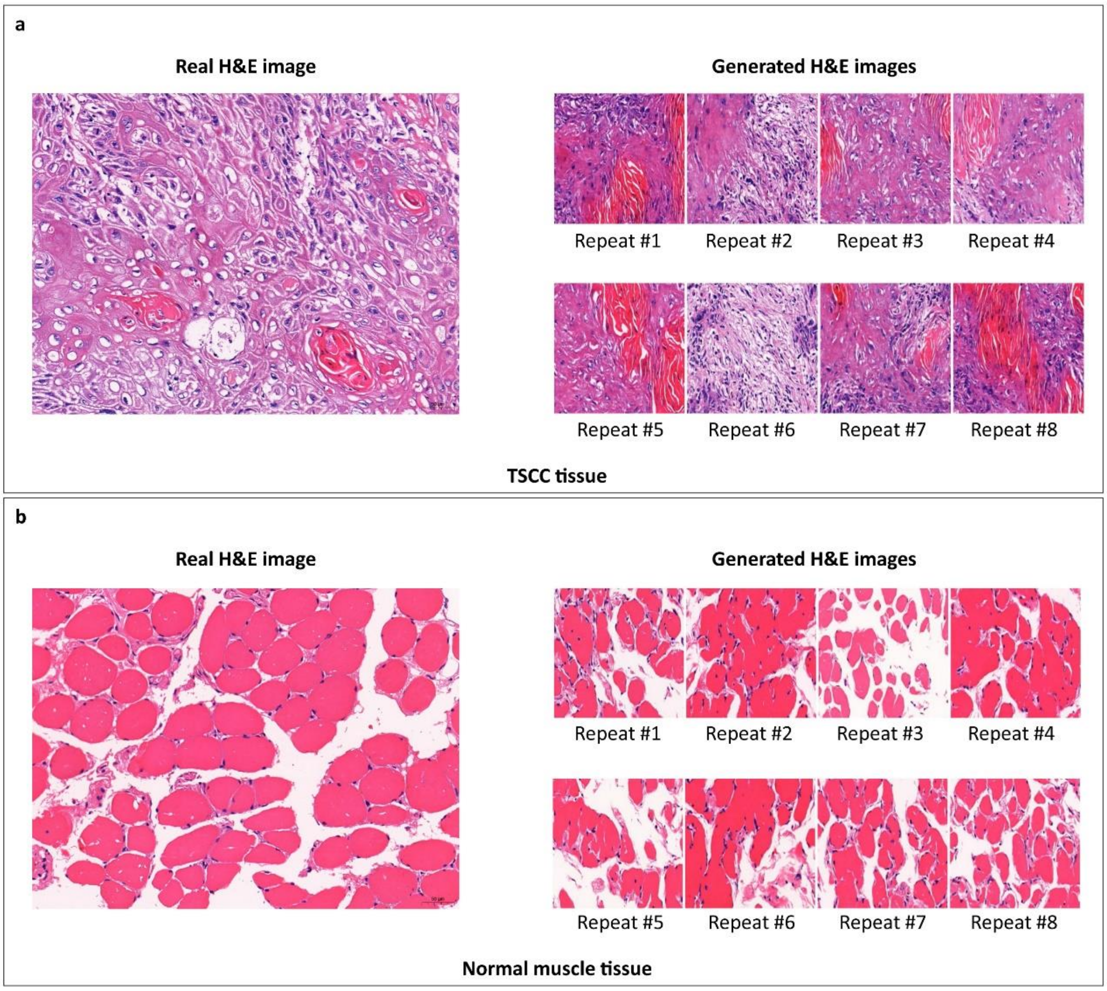

Supplementary Fig 3: Generated H&E Images from Raman Images of TSCC and Normal Tissues. Using the AI transformation model, we transformed the Raman images of TSCC and normal tissues into corresponding H&E images. As the generated images from the diffusion model slightly differ each time, we ran the model eight times for each tissue type, resulting in eight distinct H&E images. a Raman image and generated images of TSCC. From the generated images of TSCC tissue, the histopathological features essential for clinical diagnosis are clearly observed, including the pleomorphism of squamous cells, the formation of keratin pearls, and the invasion of cancer cells into the surrounding connective tissue. b Raman image and generated images of normal tissue. Similarly, in the generated images of normal tissue, the striped structure of tongue muscle cells, multiple nuclei, and connective tissue are distinctly visible.

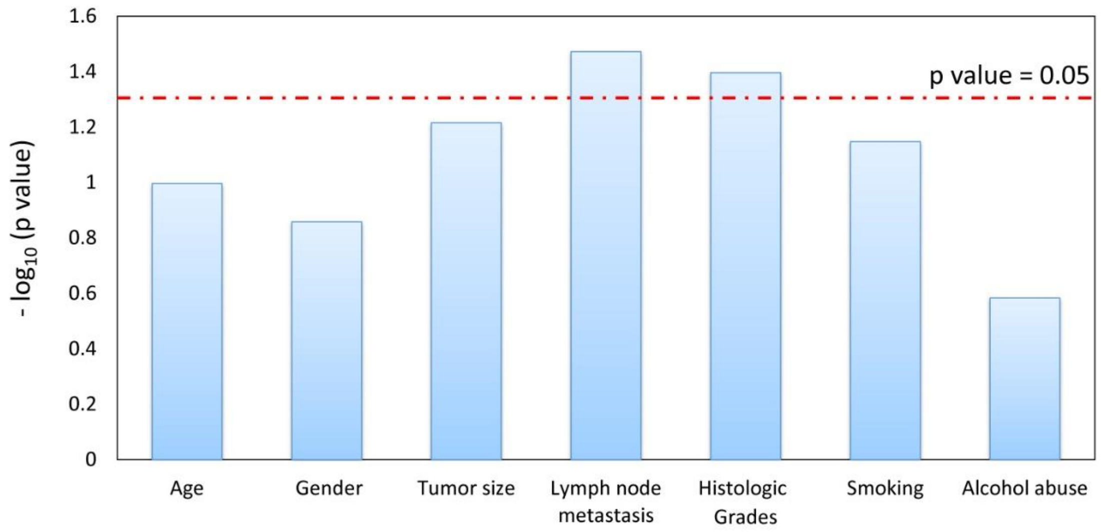

Supplementary Fig 4: Results of univariate analysis of variance on the discriminability of the generated H&E Images of TSCC by the model across various clinical indicators. The discriminability of model-generated H&E images varies significantly among TSCC samples with different lymph node metastasis and histologic grades, as well as across various clinical indicators including age, gender, smoking history and alcohol abuse.

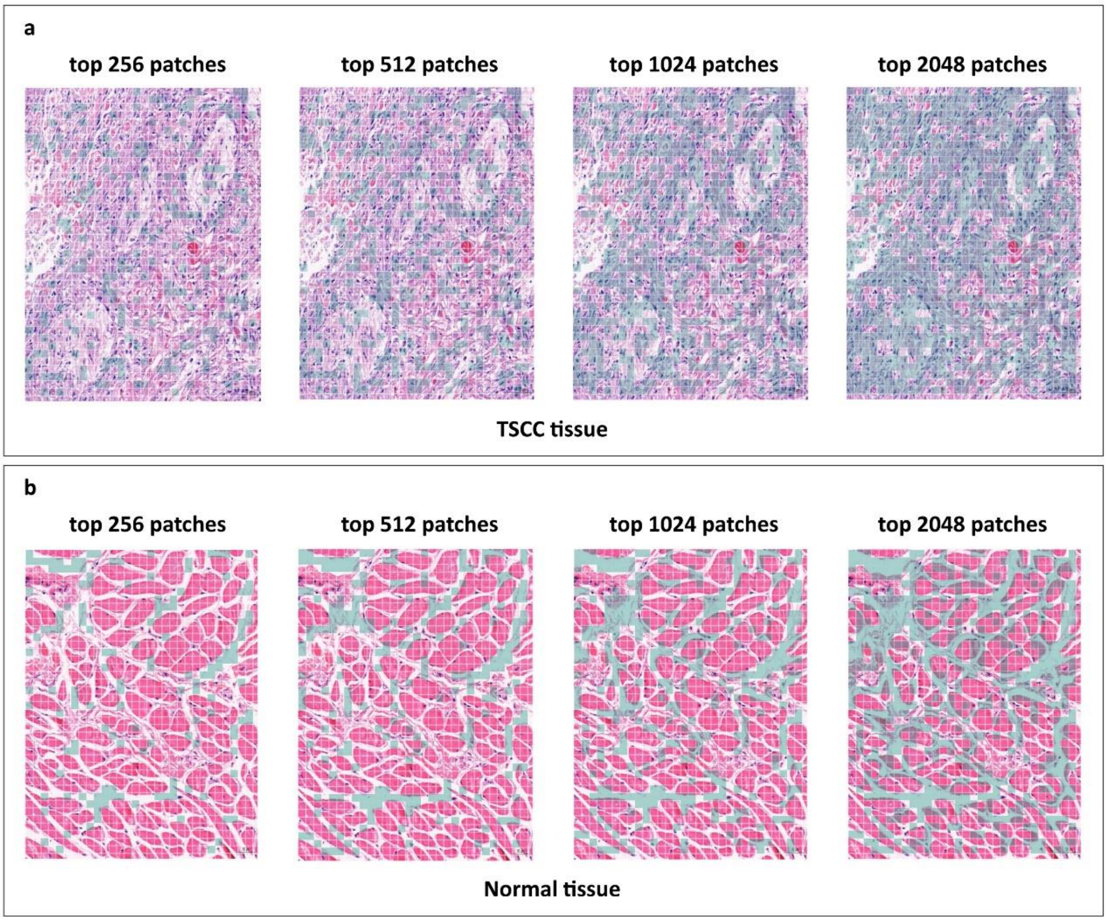

Supplementary Fig 5: **Spatial Characteristics Represented by Pre-Trained H&E Targets in the Application of the Diffusion Model for H&E Image Generation.** Pre-trained H&E targets serve as conditioning in the diffusion model to guide the generation of H&E images. We paired the pre-trained H&E targets with H&E patches, creating target-patch pairs for computing cosine similarity within each pair. Subsequently, all pairs were ranked based on their cosine similarities in descending order. The top *n* patches were then selected to assess the coverage of the real H&E image. a The coverage analysis of the top 256, 512, 1024, and 2018 patches in TSCC tissue. b The coverage analysis of the top 256, 512, 1024, and 2018 patches in normal tissue. The figure clearly exhibits the spatial characteristics captured by the pre-trained H&E targets, providing insights into the effectiveness of the diffusion model in generating H&E images with desired spatial features.

Supplementary Methods: Pseudo codes for the details of model implementation

For enhanced clarity regarding the models employed in this study, we provide pseudo code outlining the entire generation process. Given that the original code is written in Python, we've reformatted it into a process-oriented style to improve readability. In this presentation, Python's keywords, such as 'if', 'for', and 'return', are highlighted in green for easy identification. Comments are marked in blue, and functions are distinguished in orange. Variables holding multiple values are presented in **bold** for emphasis. The subscripts i, j, k and cls denote dimensions, whereas other notations specify attributes, like HE for H&E images and RA for Raman images.

Notably, as the implementation utilizes PyTorch, functions highlighted in orange but absent from the pseudo code refer to PyTorch's built-in functionalities.

$\boldsymbol{Algorithm}$ **1** $ImgAlignNet\ forward$

def forward$(\boldsymbol{Img}_{\boldsymbol{HE}_{cls}}, \boldsymbol{Img}_{\boldsymbol{RA}_{cls}}, \boldsymbol{seed}_{cls,k}, w_{HE_{cls,k}}, w_{RA_{cls,k}}, \lambda_{HE}, \lambda_{RA}, seg_{Padding}, seg_{Stride})$:

##$Parameters$:

$\boldsymbol{img}$: $the\ tensor\ of\ a\ image$

cls: $the\ class\ in\ \{TSCC, Normal\}$

HE: $H\&E\ image$

RA: $Raman\ image$

λ, w: $trainable\ parameters$

$seg_{Padding}, seg_{Stride}$: $The\ parameters\ for\ segmentation$

##$Calculating\ and\ segmenting\ the\ patches$

1: $\{\boldsymbol{Patch}_{\boldsymbol{HE}_{cls,i}}\} = \text{segImg}(\boldsymbol{Img}_{\boldsymbol{HE}_{cls}}, seg_{Padding}, seg_{Stride})$

2: $\{\boldsymbol{Patch}_{\boldsymbol{RA}_{cls,j}}\} = \text{segImg}(\boldsymbol{Img}_{\boldsymbol{RA}_{cls}}, seg_{Padding}, seg_{Stride})$

##$Generating\ targets$

3: $\{\boldsymbol{Target}_{\boldsymbol{HE}_{cls,k}}\}, \{\boldsymbol{Target}_{\boldsymbol{RA}_{cls,k}}\} = FCs(\boldsymbol{seed}_{cls,k})$

##$Computing\ the\ logits$

4: $\boldsymbol{Logits}_{\boldsymbol{HE}_{cls}} = \sum_k |w_{HE_{cls,k}}| \sum_i \exp\left(|\lambda_{HE}|(cosSimilarity(\boldsymbol{Target}_{\boldsymbol{HE}_{cls,k}}, \boldsymbol{Patch}_{\boldsymbol{HE}_{cls,i}}) + 1)\right)$

5: $\boldsymbol{Logits}_{\boldsymbol{RA}_{cls}} = \sum_k |w_{RA_{cls,k}}| \sum_j \exp\left(|\lambda_{RA}|\left(cosSimilarity\left(\boldsymbol{Target}_{\boldsymbol{RA}_{cls,k}}, \boldsymbol{Patch}_{\boldsymbol{RA}_{cls,j}}\right) + 1\right)\right)$

##$Computing\ loss\ value\ using\ cross\ entrophy$

6: $Loss = CE(\text{softmax}_{cls}(\boldsymbol{Logits}_{\boldsymbol{RA}}), cls) + CE(\text{softmax}_{cls}(\boldsymbol{Logits}_{\boldsymbol{HE}}), cls)$

$return$ $\text{Loss}, \{\boldsymbol{Target}_{\boldsymbol{HE}_{cls,k}}\}, \{\boldsymbol{Target}_{\boldsymbol{RA}_{cls,k}}\}$

Algorithm 2 *ImgAlignModel segment image*

def segImg($\boldsymbol{img}, seg_{Padding}, seg_{Stride}$):

##Parameters:

  $\boldsymbol{img}$: *the tensor of a image*

  $seg_{Padding}, seg_{Stride}$: *The parameters for segmentation*

##Downsampling the image

1: $\boldsymbol{img} = \text{CNNDownSampling}(\boldsymbol{img})$

##Segmenting the image

2: $\{\boldsymbol{Patch_i}\} = \text{unfold}(\boldsymbol{img}, seg_{Padding}, seg_{Stride})$

##Adjust the dimension for every patch using a fully connected layer

3: $\boldsymbol{Patch_i} = \text{FC}(\boldsymbol{Patch_i})$

##Normalizing

4: $\boldsymbol{Patch_i} = \text{normalize}(p = 2)$

return $\{\boldsymbol{Patch_i}\}$

Algorithm 3 *CNN down sampling block*

def CNNDownSampling($\boldsymbol{x}, N_{iter} = 2$):

##Parameters:

  $\boldsymbol{x}$: *the input tensor*

  N_{iter}: *the number of iteration*

##Initial convolution

1: $\boldsymbol{x} = \text{Conv2d}(\boldsymbol{x}, kernelSize = 1)$

2: *for* $n \in \{1 \ldots N_{iter}\}$:

3:   $\boldsymbol{x} = \text{resBlock}(\boldsymbol{x})$

##The down operation is processed by Conv2d

4:   $\boldsymbol{x} = \text{Conv2d}(\boldsymbol{x}, kernelSize = 3, stride = 2, padding = 1)$

5:   $\boldsymbol{x} = \text{resBlock}(\boldsymbol{x})$

return $\boldsymbol{x}$

Algorithm 4 *residule block*

def resBlock($\boldsymbol{x}$):

##Parameters:

  $\boldsymbol{x}$: *the input tensor*

##The two convolution layers below uses $kernelSize = 3, stride = 1, padding = 1$

1: $\boldsymbol{h} = \text{Conv2d}\left(\text{SiLU}\left(\text{GroupNorm}(\boldsymbol{x})\right)\right)$

2: $\boldsymbol{h} = \text{Conv2d}\left(\text{Dropout}\left(\text{SiLU}\left(\text{GroupNorm}(\boldsymbol{h})\right)\right)\right)$

##Ensure the number of channels of shortcut is the same

3: *if* $\boldsymbol{dim}(\boldsymbol{h}) \neq \boldsymbol{dim}(\boldsymbol{x})$:

##The convolution layer below uses $kernelSize = 1$

  $\boldsymbol{x} = \text{Conv2d}(\boldsymbol{x})$

return $\boldsymbol{h} + \boldsymbol{x}$

Algorithm 5 *the full connection layers for generating targets*

def FCs($\boldsymbol{seed}, N_{iter} = 2$):

##Parameters:

  $\boldsymbol{seed}$: *the seed tensor generated from a standard normal distribution*

  N_{iter}: *the number of iteration*

$\boldsymbol{Target_{HE}} = \mathrm{FC}(\boldsymbol{x})$

$\boldsymbol{Target_{RA}} = \mathrm{FC}(\boldsymbol{x})$

for $n \in \{1 \ldots N_{iter}\}$:

  $\boldsymbol{Target_{HE}} = \mathrm{FC}\left(\mathrm{SiLU}\left(\mathrm{LayerNorm}(\boldsymbol{Target_{HE}})\right)\right)$

  $\boldsymbol{Target_{RA}} = \mathrm{FC}\left(\mathrm{SiLU}\left(\mathrm{LayerNorm}(\boldsymbol{Target_{RA}})\right)\right)$

return $\boldsymbol{Target_{HE}}, \boldsymbol{Target_{RA}}$

Algorithm 6 *Diffusion Model training process*

def *fit*($\boldsymbol{Img_{HE_{cls}}}, \{\boldsymbol{Target_{HE_{cls,k}}}\}$):

##Parameters:

  $\boldsymbol{img}$: *the tensor of a image*

  $\boldsymbol{Target}$: *the optimized parameter from ImgAlignNet*

  cls: the class in {TSCC, Normal}

  HE: H&E image

##Generating noise from a standard normal distribution

$\boldsymbol{noise} = \mathrm{randn\_like}\left(\boldsymbol{Img_{HE_{cls}}}\right)$

##Adding noise, also called q sampling process: $q(x_t|x_0)$

$\boldsymbol{Img'_{HE_{cls}}}, t = \mathrm{qSample}\left(\boldsymbol{Img_{HE_{cls}}}, \boldsymbol{noise}\right)$

##Using UNet for fitting the noise

$\boldsymbol{rec_{noise}} = \mathrm{diffsionUNet}\left(\boldsymbol{Img'_{HE_{cls}}}, t, \{\boldsymbol{Target_{HE_{cls,k}}}\}\right)$

$Loss = \mathrm{MSE}(\boldsymbol{noise}, \boldsymbol{rec_{noise}})$

return *Loss*

Algorithm 7 *Diffusion Model inference process*

def *inference*($\boldsymbol{Img_{RA}}, \boldsymbol{shape_{img}}$):

##Parameters:

  $\boldsymbol{img}$: *the tensor of a image*

  $\boldsymbol{shape}$: *the shape of the image to generate*

  RA: Raman image

##Generating the conditioning

$\{\boldsymbol{cond_k}\} = \mathrm{calcCond}\left(\boldsymbol{Img_{RA_{cls}}}\right)$

##Generating noise from a standard normal distribution

$\boldsymbol{noise} = \mathrm{randn}\left(\boldsymbol{shape_{img}}\right)$

##The reverse loop, also called p sampling process $p(x_{t-1}|x_t)$

$\boldsymbol{rec} = \mathrm{pSample}(\boldsymbol{noise}, \{\boldsymbol{cond_k}\})$

return $\boldsymbol{rec}$

***Algorithm* 8** *The q sampling process*

def qSample($\boldsymbol{Img_{HE_{cls}}}, \boldsymbol{noise}, t_{Num} = 1000, \boldsymbol{\beta_{init}} = [0.0001,0.02]$):

##Parameters:

  img: *the tensor of a image*

  cls: *the class in {TSCC, Normal}*

  HE: *H&E image*

  noise: *the noise from N(0,1) with the same shape of* ***img***

  t_{Num}: the number of diffusion steps

  $\boldsymbol{\beta}_{\mathbf{init}}$: a hyper parameter *contain the start and end value of* β

##Randomly generate a step with in t_{Num}

1: t = randint($0, t_{Num}$)

##Abstracting necessary parameter

2: $\tilde{\alpha}_t$ = calcAlphaCumprod($t, t_{Num}, \boldsymbol{\beta_{init}}$)

##Adding the noise

3: $\boldsymbol{Img'_{HE_{cls}}} = \sqrt{\tilde{\alpha}_t}\boldsymbol{Img_{HE_{cls}}} + \sqrt{1-\tilde{\alpha}_t}\boldsymbol{noise}$

return *Loss*

***Algorithm* 9** *Calculating cumulative product of* α

def calcAlphaCumprod($t, t_{Num} = 1000, \boldsymbol{\beta_{init}} = [0.0001,0.02]$):

##Parameters:

  t: a time step

  t_{Num}: the number of diffusion steps

  $\boldsymbol{\beta}_{\mathbf{init}}$: a hyper parameter *contain the start and end value of* β

##Generating $\boldsymbol{\beta}$ *using linear schedule*

1: $\beta_{start}, \beta_{end} = \boldsymbol{\beta_{init}}$

2: $\boldsymbol{\beta}$ = linspace($\beta_{start}, \beta_{end}, t_{Num}$)

##Computing $\boldsymbol{\alpha}$ *and* $\tilde{\boldsymbol{\alpha}}$*, generally they are global values*

3: $\boldsymbol{\alpha} = 1 - \boldsymbol{\beta}$

4: $\tilde{\boldsymbol{\alpha}}$ = cumprod($\boldsymbol{\alpha}$)

## only return the tth *value*

return $\tilde{\alpha}_t$

$\boldsymbol{Algorithm}$ **10** *Computing the conditioning information*

def calcCond$(\boldsymbol{Img_{RA}}, \{\boldsymbol{Target_{HE_{cls,k}}}\}, \{\boldsymbol{Target_{RA_{cls,k}}}\}, \lambda_{RA}, seg_{Padding}, seg_{Stride}, k_t = 2, k_p = 4)$:

##*Parameters*:

$\boldsymbol{noise}$: *the tensor of noise*

$cond$: *the conditions*

$\{\boldsymbol{Target_{HE_{cls,k}}}\}, \{\boldsymbol{Target_{RA_{cls,k}}}\}$: The optimized targets

λ_{RA}: *the optimized parameter of ImgAlignNet*

$seg_{Padding}, seg_{Stride}$: *The parameters for segmentation*

k_t, k_p: the parameter for topK operation

##*Segmenting the Raman image*

1: $\{\boldsymbol{Patch_{RA_i}}\} =$ segImg$(\boldsymbol{Img_{RA}}, seg_{Padding}, seg_{Stride})$

##*Computing the cosine similarity*

2: $\boldsymbol{dist_{cls,k,i}} = \exp\left(|\lambda_{RA}|\left(\text{cosSimilarity}\left(\boldsymbol{Target_{RA_{cls,k}}}, \boldsymbol{Patch_{RA_i}}\right) + 1\right)\right)$

##*Using topK operation for filtering small values*

3: $\boldsymbol{dist_{k',i}} =$ reshape$(\boldsymbol{dist_{cls,k,i}}, [-\boldsymbol{1}, \boldsymbol{i}])$

4: $\boldsymbol{dist_{k',i'}} =$ topk$(\boldsymbol{dist_{k',i}}, k_p, dim = -1)$

##*The second topK operation only abstract the values as threshold*

5: $thres =$ topk$(\boldsymbol{dist_{k',i'}}, k_t, dim = 1)$. values

6: *if* $dist_{k',i'} <$ thres:

7: $\quad dist_{k',i'} = -\inf$

8: $\boldsymbol{dist_{cls,k,i'}} =$ reshape$(\text{softmax}_{k'}(\boldsymbol{dist_{k',i'}}), [\boldsymbol{cls}, -\boldsymbol{1}, \boldsymbol{i'}])$

##*Using attention – like operation generating conditioning*

##*Note that the target changes to the corresponding HE targets*

9: $\mathbf{cond_{i'}} = \sum_{cls,k} \boldsymbol{dist_{cls,k,i'}} \boldsymbol{Target_{HE_{cls,k}}}$

return $\{\mathbf{cond_{i'}}\}$

Algorithm 11 *The p sampling process*

def pSample($\boldsymbol{noise}, \{\boldsymbol{cond_k}\}, t_{Num} = 1000$):

##Parameters:

$\boldsymbol{noise}$: *the tensor of noise, representing* x_t

cond: the conditions

t_{Num}: *the number of diffusion steps*

1: *for* $t \in \{1, \ldots, t_{Num}\}$:

##Using UNet to compute the noise ϵ *for last step:*

2: $\boldsymbol{pred} =$ diffsionUNet($\boldsymbol{noise}, t, \{\boldsymbol{cond_k}\}$)

##Generating necessary parameters, the $\boldsymbol{\beta_{init}}$ *is globally consistent:*

3: $\tilde{\alpha}_t =$ calcAlphaCumprod($t, t_{Num}, \boldsymbol{\beta_{init}}$)

##Computing x_0 *from* $x_t(x_0, \epsilon) = \sqrt{\tilde{\alpha}_t} x_0 + \sqrt{1 - \tilde{\alpha}_t} \epsilon$:

4: $\boldsymbol{pred'} = \sqrt{\frac{1}{\tilde{\alpha}_t}} \boldsymbol{noise} - \sqrt{\frac{1 - \tilde{\alpha}_t}{\tilde{\alpha}_t}} \boldsymbol{pred}$

##Computing posterior values:

5: $\boldsymbol{\mu_t}, \boldsymbol{\sigma}_t =$ calcPosteriorVals($\boldsymbol{pred'}, \boldsymbol{noise}, t, t_{Num}, \boldsymbol{\beta_{init}}$)

6: *if* $t > 1$:

7: $\boldsymbol{noise} = \boldsymbol{\mu_t} +$ randn_like($\boldsymbol{noise}$)$\boldsymbol{\sigma}_t$

8: *else*:

9: $\boldsymbol{noise} = \boldsymbol{\mu_t}$

return $\boldsymbol{noise}$

Algorithm 12 *Calculating the posterior values*

```
def calcPosteriorVals(pred', noise, t, t_Num = 1000, β_init = [0.0001,0.02]):
##Parameters:
    pred', noise: the input tensors from Algorithm 11 represent x_0 and x_t
    t: a time step
    t_Num: the number of diffusion steps
    β_init: a hyper parameter contain the start and end value of β
##Generating β using linear schedule
1: β_start, β_end = β_init
2: β = linspace(β_start, β_end, t_Num)
##Computing α and α̃, generally they are global values
3: α = 1 − β
4: α̃ = cumprod(α)
##Generating a α̃_prev, representing the previous step of α̃
5: α̃_prev_t = α̃_{t−1}, α̃_prev_1 = 0
##Computing the posterior values from q(x_{t−1}|x_t, x_0) = q(x_t|x_{t−1}, x_0) q(x_{t−1}|x_0) / q(x_t|x_0)
6: μ = β·sqrt(α̃_prev)/(1 − α̃) · pred' + (1 − α̃_prev)·sqrt(α)/(1 − α̃) · noise
7: σ = sqrt( β(1 − α̃_prev) / (1 − α̃) )
## only return the t^th value
return μ_t, σ_t
```

Where:

$$q(x_{t-1}|x_t,x_0)=q(x_t|x_{t-1},x_0)\frac{q(x_{t-1}|x_0)}{q(x_t|x_0)}$$

$$\boldsymbol{\mu}=\frac{\boldsymbol{\beta}\sqrt{\tilde{\boldsymbol{\alpha}}_{prev}}}{1-\tilde{\boldsymbol{\alpha}}}\boldsymbol{pred'}+\frac{(1-\tilde{\boldsymbol{\alpha}}_{prev})\sqrt{\boldsymbol{\alpha}}}{1-\tilde{\boldsymbol{\alpha}}}\boldsymbol{noise}$$

$$\boldsymbol{\sigma}=\sqrt{\frac{\boldsymbol{\beta}(1-\tilde{\boldsymbol{\alpha}}_{prev})}{1-\tilde{\boldsymbol{\alpha}}}}$$

***Algorithm* 13** *the forward process of Diffusion UNet*

def diffusionUNet($\boldsymbol{x}, t, \boldsymbol{cond}, n_{iter} = 5, attStartIdx = 4$):

##Parameters:

  $\boldsymbol{x}$: *the input tensor*

  t: *a time step*

  $\boldsymbol{cond}$: *the conditioning information*

  n_{iter}: *the number of iteration*

  $attStartIdx$: *the index where start to use attention*

##Embeding time step, the position encoding method is from transformer

$\boldsymbol{t_{ebd}} = \mathrm{FC}\left(\mathrm{SiLU}\left(\mathrm{FC}(\mathrm{pos\_encoding}(t))\right)\right)$

$\boldsymbol{h_1} = \boldsymbol{x}$

##Down sampling iterations

for $n \in \{1 \dots n_{iter}\}$:

  $\boldsymbol{h_{n+1}} = \mathrm{resBlock}\left(\mathrm{resBlock}(\boldsymbol{h_n})\right)$

  if $n \geq attStartIdx$:

    $\boldsymbol{h_{n+1}} = \mathrm{crossAttention}(\mathrm{selfAttention}(\boldsymbol{h_{n+1}}), \boldsymbol{cond})$

##Down sampling layer implemented by convolution layer

  $\boldsymbol{h_{n+1}} = \mathrm{Conv2d}(\boldsymbol{h_{n+1}}, kernelSize = 3, stride = 2, padding = 1)$

##After the iteration of down sampling, the footnote should be $n_{iter} + 1$

$\boldsymbol{g_{n_{iter}+1}} = \mathrm{resBlock}\left(\mathrm{crossAttention}\left(\mathrm{selfAttention}\left(\left(\mathrm{resBlock}(\boldsymbol{h_{n_{iter}+1}})\right)\right), \boldsymbol{cond}\right)\right)$

##Up sampling iterations

for $n \in \{n_{iter} \dots 1\}$:

  $\boldsymbol{g_n} = \mathrm{resBlock}\left(\mathrm{resBlock}(\mathrm{cat}(\boldsymbol{g_{n+1}}, \boldsymbol{h_{n+1}}))\right)$

  if $n \geq attStartIdx$:

    $\boldsymbol{g_n} = \mathrm{crossAttention}(\mathrm{selfAttention}(\boldsymbol{g_n}), \boldsymbol{cond})$

##Up sampling layer implemented by interpolate operation

  $\boldsymbol{g_n} = \mathrm{interpolate}(\boldsymbol{h_n}, scale = 2)$

$\boldsymbol{img} = \mathrm{Conv2d}(\mathrm{SiLU}(\mathrm{GroupNorm}(\boldsymbol{g_1})))$

return $\boldsymbol{img}$

Supplementary Table 1: Demographic information for the TSCC patients

Case information		Patients (%)
All patients		15(100)
Age	Range from 29 to 79 years	
	Median 67 years	
Gender	Female	4(26.7%)
	Male	11(73.3%)
Tumor size	T1	3(20%)
	T2	9(60%)
	T3	1(6.7%)
	T4	2(13.3%)
Lymph node metastasis	Positive	9(60%)
	Negative	6(40%)
Smoking	Positive	6(40%)
	Negative	9(60%)
Alcohol abuse	Positive	5(33.3%)
	Negative	10(66.7%)

Supplementary Table 2: Independently classified results for generated H&E images by three pathologists.

TSCC Sample #1

Generated H&E images	Pathologist #1	Pathologist #2	Pathologist #3
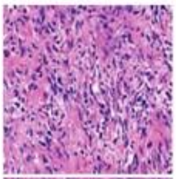	☑ TSCC<br>☐ Normal muscle tissue<br>☐ Not sure	☑ TSCC<br>☐ Normal muscle tissue<br>☐ Not sure	☑ TSCC<br>☐ Normal muscle tissue<br>☐ Not sure
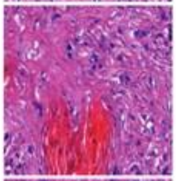	☑ TSCC<br>☐ Normal muscle tissue<br>☐ Not sure	☑ TSCC<br>☐ Normal muscle tissue<br>☐ Not sure	☑ TSCC<br>☐ Normal muscle tissue<br>☐ Not sure
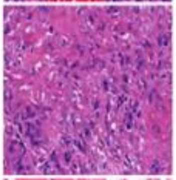	☑ TSCC<br>☐ Normal muscle tissue<br>☐ Not sure	☑ TSCC<br>☐ Normal muscle tissue<br>☐ Not sure	☑ TSCC<br>☐ Normal muscle tissue<br>☐ Not sure
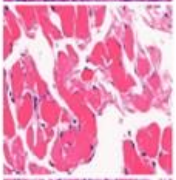	☐ TSCC<br>☑ Normal muscle tissue<br>☐ Not sure	☐ TSCC<br>☑ Normal muscle tissue<br>☐ Not sure	☐ TSCC<br>☑ Normal muscle tissue<br>☐ Not sure
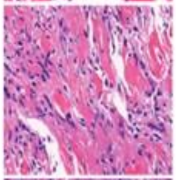	☐ TSCC<br>☑ Normal muscle tissue<br>☐ Not sure	☐ TSCC<br>☐ Normal muscle tissue<br>☑ Not sure	☐ TSCC<br>☑ Normal muscle tissue<br>☐ Not sure
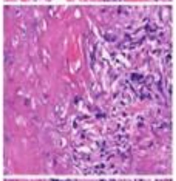	☑ TSCC<br>☐ Normal muscle tissue<br>☐ Not sure	☑ TSCC<br>☐ Normal muscle tissue<br>☐ Not sure	☑ TSCC<br>☐ Normal muscle tissue<br>☐ Not sure
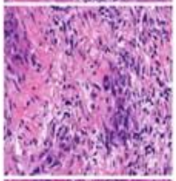	☑ TSCC<br>☐ Normal muscle tissue<br>☐ Not sure	☑ TSCC<br>☐ Normal muscle tissue<br>☐ Not sure	☑ TSCC<br>☐ Normal muscle tissue<br>☐ Not sure
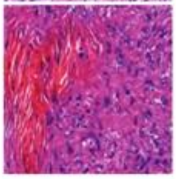	☑ TSCC<br>☐ Normal muscle tissue<br>☐ Not sure	☑ TSCC<br>☐ Normal muscle tissue<br>☐ Not sure	☑ TSCC<br>☐ Normal muscle tissue<br>☐ Not sure

TSCC Sample #2

Generated H&E images	Pathologist #1	Pathologist #2	Pathologist #3
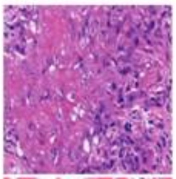	☑ TSCC<br>☐ Normal muscle tissue<br>☐ Not sure	☑ TSCC<br>☐ Normal muscle tissue<br>☐ Not sure	☑ TSCC<br>☐ Normal muscle tissue<br>☐ Not sure
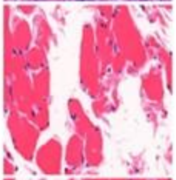	☐ TSCC<br>☑ Normal muscle tissue<br>☐ Not sure	☐ TSCC<br>☑ Normal muscle tissue<br>☐ Not sure	☐ TSCC<br>☑ Normal muscle tissue<br>☐ Not sure
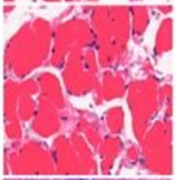	☐ TSCC<br>☑ Normal muscle tissue<br>☐ Not sure	☐ TSCC<br>☑ Normal muscle tissue<br>☐ Not sure	☐ TSCC<br>☑ Normal muscle tissue<br>☐ Not sure
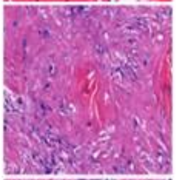	☑ TSCC<br>☐ Normal muscle tissue<br>☐ Not sure	☑ TSCC<br>☐ Normal muscle tissue<br>☐ Not sure	☑ TSCC<br>☐ Normal muscle tissue<br>☐ Not sure
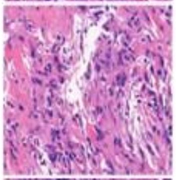	☐ TSCC<br>☐ Normal muscle tissue<br>☑ Not sure	☐ TSCC<br>☐ Normal muscle tissue<br>☑ Not sure	☑ TSCC<br>☐ Normal muscle tissue<br>☐ Not sure
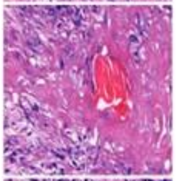	☑ TSCC<br>☐ Normal muscle tissue<br>☐ Not sure	☑ TSCC<br>☐ Normal muscle tissue<br>☐ Not sure	☑ TSCC<br>☐ Normal muscle tissue<br>☐ Not sure
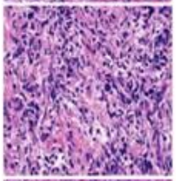	☑ TSCC<br>☐ Normal muscle tissue<br>☐ Not sure	☑ TSCC<br>☐ Normal muscle tissue<br>☐ Not sure	☑ TSCC<br>☐ Normal muscle tissue<br>☐ Not sure
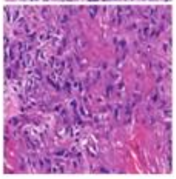	☑ TSCC<br>☐ Normal muscle tissue<br>☐ Not sure	☑ TSCC<br>☐ Normal muscle tissue<br>☐ Not sure	☑ TSCC<br>☐ Normal muscle tissue<br>☐ Not sure

TSCC Sample #3

Generated H&E images	Pathologist #1	Pathologist #2	Pathologist #3
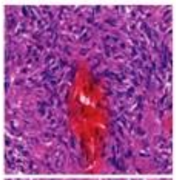	☑ TSCC<br>☐ Normal muscle tissue<br>☐ Not sure	☑ TSCC<br>☐ Normal muscle tissue<br>☐ Not sure	☑ TSCC<br>☐ Normal muscle tissue<br>☐ Not sure
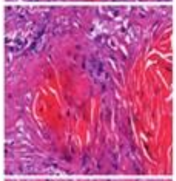	☑ TSCC<br>☐ Normal muscle tissue<br>☐ Not sure	☑ TSCC<br>☐ Normal muscle tissue<br>☐ Not sure	☑ TSCC<br>☐ Normal muscle tissue<br>☐ Not sure
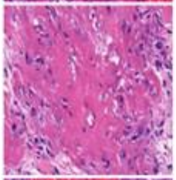	☑ TSCC<br>☐ Normal muscle tissue<br>☐ Not sure	☑ TSCC<br>☐ Normal muscle tissue<br>☐ Not sure	☑ TSCC<br>☐ Normal muscle tissue<br>☐ Not sure
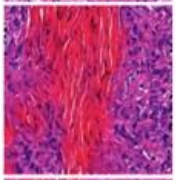	☑ TSCC<br>☐ Normal muscle tissue<br>☐ Not sure	☑ TSCC<br>☐ Normal muscle tissue<br>☐ Not sure	☑ TSCC<br>☐ Normal muscle tissue<br>☐ Not sure
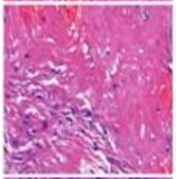	☐ TSCC<br>☐ Normal muscle tissue<br>☑ Not sure	☑ TSCC<br>☐ Normal muscle tissue<br>☐ Not sure	☑ TSCC<br>☐ Normal muscle tissue<br>☐ Not sure
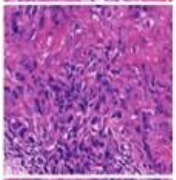	☑ TSCC<br>☐ Normal muscle tissue<br>☐ Not sure	☑ TSCC<br>☐ Normal muscle tissue<br>☐ Not sure	☑ TSCC<br>☐ Normal muscle tissue<br>☐ Not sure
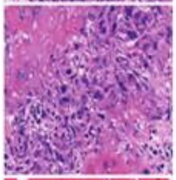	☑ TSCC<br>☐ Normal muscle tissue<br>☐ Not sure	☑ TSCC<br>☐ Normal muscle tissue<br>☐ Not sure	☑ TSCC<br>☐ Normal muscle tissue<br>☐ Not sure
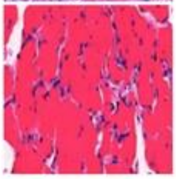	☐ TSCC<br>☑ Normal muscle tissue<br>☐ Not sure	☐ TSCC<br>☑ Normal muscle tissue<br>☐ Not sure	☐ TSCC<br>☑ Normal muscle tissue<br>☐ Not sure

TSCC Sample #4

Generated H&E images	Pathologist #1	Pathologist #2	Pathologist #3
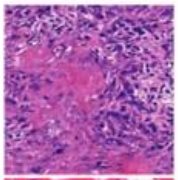	☑ TSCC<br>☐ Normal muscle tissue<br>☐ Not sure	☑ TSCC<br>☐ Normal muscle tissue<br>☐ Not sure	☑ TSCC<br>☐ Normal muscle tissue<br>☐ Not sure
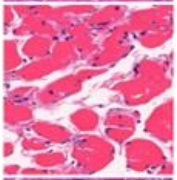	☐ TSCC<br>☑ Normal muscle tissue<br>☐ Not sure	☐ TSCC<br>☑ Normal muscle tissue<br>☐ Not sure	☐ TSCC<br>☑ Normal muscle tissue<br>☐ Not sure
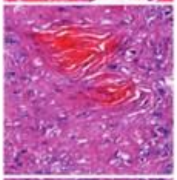	☑ TSCC<br>☐ Normal muscle tissue<br>☐ Not sure	☑ TSCC<br>☐ Normal muscle tissue<br>☐ Not sure	☑ TSCC<br>☐ Normal muscle tissue<br>☐ Not sure
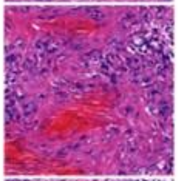	☑ TSCC<br>☐ Normal muscle tissue<br>☐ Not sure	☑ TSCC<br>☐ Normal muscle tissue<br>☐ Not sure	☑ TSCC<br>☐ Normal muscle tissue<br>☐ Not sure
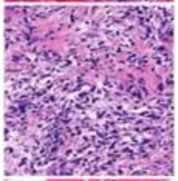	☑ TSCC<br>☐ Normal muscle tissue<br>☐ Not sure	☑ TSCC<br>☐ Normal muscle tissue<br>☐ Not sure	☑ TSCC<br>☐ Normal muscle tissue<br>☐ Not sure
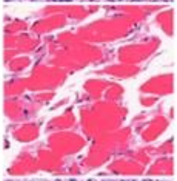	☐ TSCC<br>☑ Normal muscle tissue<br>☐ Not sure	☐ TSCC<br>☑ Normal muscle tissue<br>☐ Not sure	☐ TSCC<br>☑ Normal muscle tissue<br>☐ Not sure
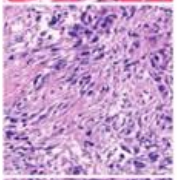	☑ TSCC<br>☐ Normal muscle tissue<br>☐ Not sure	☑ TSCC<br>☐ Normal muscle tissue<br>☐ Not sure	☑ TSCC<br>☐ Normal muscle tissue<br>☐ Not sure
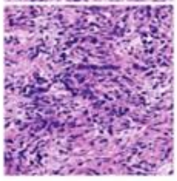	☑ TSCC<br>☐ Normal muscle tissue<br>☐ Not sure	☑ TSCC<br>☐ Normal muscle tissue<br>☐ Not sure	☑ TSCC<br>☐ Normal muscle tissue<br>☐ Not sure

TSCC Sample #5

Generated H&E images	Pathologist #1	Pathologist #2	Pathologist #3
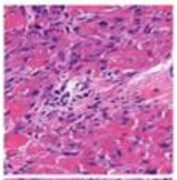	☑ TSCC<br>☐ Normal muscle tissue<br>☐ Not sure	☑ TSCC<br>☐ Normal muscle tissue<br>☐ Not sure	☑ TSCC<br>☐ Normal muscle tissue<br>☐ Not sure
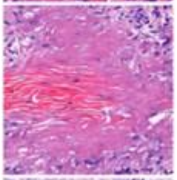	☑ TSCC<br>☐ Normal muscle tissue<br>☐ Not sure	☑ TSCC<br>☐ Normal muscle tissue<br>☐ Not sure	☑ TSCC<br>☐ Normal muscle tissue<br>☐ Not sure
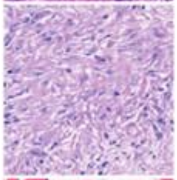	☑ TSCC<br>☐ Normal muscle tissue<br>☐ Not sure	☐ TSCC<br>☐ Normal muscle tissue<br>☑ Not sure	☑ TSCC<br>☐ Normal muscle tissue<br>☐ Not sure
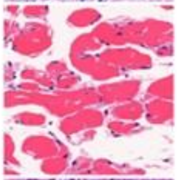	☐ TSCC<br>☑ Normal muscle tissue<br>☐ Not sure	☐ TSCC<br>☑ Normal muscle tissue<br>☐ Not sure	☐ TSCC<br>☑ Normal muscle tissue<br>☐ Not sure
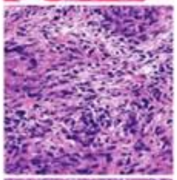	☑ TSCC<br>☐ Normal muscle tissue<br>☐ Not sure	☑ TSCC<br>☐ Normal muscle tissue<br>☐ Not sure	☑ TSCC<br>☐ Normal muscle tissue<br>☐ Not sure
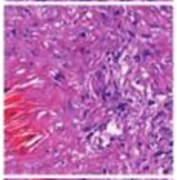	☑ TSCC<br>☐ Normal muscle tissue<br>☐ Not sure	☑ TSCC<br>☐ Normal muscle tissue<br>☐ Not sure	☑ TSCC<br>☐ Normal muscle tissue<br>☐ Not sure
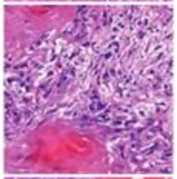	☑ TSCC<br>☐ Normal muscle tissue<br>☐ Not sure	☑ TSCC<br>☐ Normal muscle tissue<br>☐ Not sure	☑ TSCC<br>☐ Normal muscle tissue<br>☐ Not sure
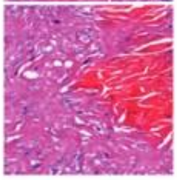	☐ TSCC<br>☐ Normal muscle tissue<br>☑ Not sure	☑ TSCC<br>☐ Normal muscle tissue<br>☐ Not sure	☑ TSCC<br>☐ Normal muscle tissue<br>☐ Not sure

TSCC Sample #6

Generated H&E images	Pathologist #1	Pathologist #2	Pathologist #3
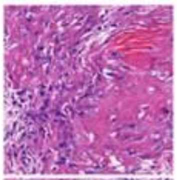	☑ TSCC<br>☐ Normal muscle tissue<br>☐ Not sure	☑ TSCC<br>☐ Normal muscle tissue<br>☐ Not sure	☑ TSCC<br>☐ Normal muscle tissue<br>☐ Not sure
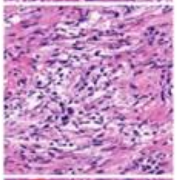	☑ TSCC<br>☐ Normal muscle tissue<br>☐ Not sure	☐ TSCC<br>☐ Normal muscle tissue<br>☑ Not sure	☑ TSCC<br>☐ Normal muscle tissue<br>☐ Not sure
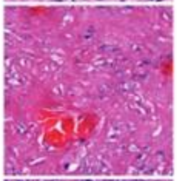	☑ TSCC<br>☐ Normal muscle tissue<br>☐ Not sure	☑ TSCC<br>☐ Normal muscle tissue<br>☐ Not sure	☑ TSCC<br>☐ Normal muscle tissue<br>☐ Not sure
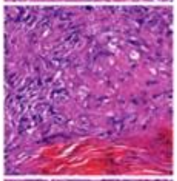	☑ TSCC<br>☐ Normal muscle tissue<br>☐ Not sure	☑ TSCC<br>☐ Normal muscle tissue<br>☐ Not sure	☑ TSCC<br>☐ Normal muscle tissue<br>☐ Not sure
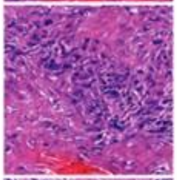	☑ TSCC<br>☐ Normal muscle tissue<br>☐ Not sure	☑ TSCC<br>☐ Normal muscle tissue<br>☐ Not sure	☑ TSCC<br>☐ Normal muscle tissue<br>☐ Not sure
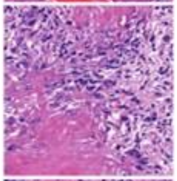	☑ TSCC<br>☐ Normal muscle tissue<br>☐ Not sure	☑ TSCC<br>☐ Normal muscle tissue<br>☐ Not sure	☑ TSCC<br>☐ Normal muscle tissue<br>☐ Not sure
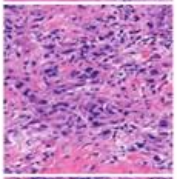	☑ TSCC<br>☐ Normal muscle tissue<br>☐ Not sure	☑ TSCC<br>☐ Normal muscle tissue<br>☐ Not sure	☑ TSCC<br>☐ Normal muscle tissue<br>☐ Not sure
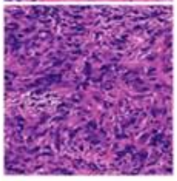	☑ TSCC<br>☐ Normal muscle tissue<br>☐ Not sure	☑ TSCC<br>☐ Normal muscle tissue<br>☐ Not sure	☑ TSCC<br>☐ Normal muscle tissue<br>☐ Not sure

TSCC Sample #7

Generated H&E images	Pathologist #1	Pathologist #2	Pathologist #3
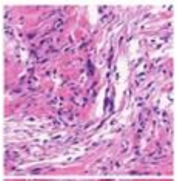	☑ TSCC<br>☐ Normal muscle tissue<br>☐ Not sure	☑ TSCC<br>☐ Normal muscle tissue<br>☐ Not sure	☑ TSCC<br>☐ Normal muscle tissue<br>☐ Not sure
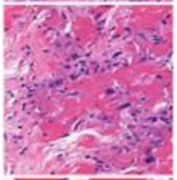	☑ TSCC<br>☐ Normal muscle tissue<br>☐ Not sure	☐ TSCC<br>☐ Normal muscle tissue<br>☑ Not sure	☑ TSCC<br>☐ Normal muscle tissue<br>☐ Not sure
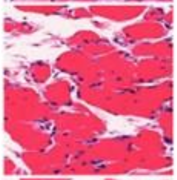	☐ TSCC<br>☑ Normal muscle tissue<br>☐ Not sure	☐ TSCC<br>☑ Normal muscle tissue<br>☐ Not sure	☐ TSCC<br>☑ Normal muscle tissue<br>☐ Not sure
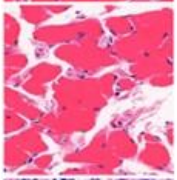	☐ TSCC<br>☑ Normal muscle tissue<br>☐ Not sure	☐ TSCC<br>☑ Normal muscle tissue<br>☐ Not sure	☐ TSCC<br>☑ Normal muscle tissue<br>☐ Not sure
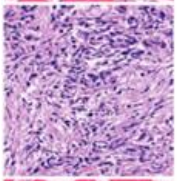	☑ TSCC<br>☐ Normal muscle tissue<br>☐ Not sure	☑ TSCC<br>☐ Normal muscle tissue<br>☐ Not sure	☑ TSCC<br>☐ Normal muscle tissue<br>☐ Not sure
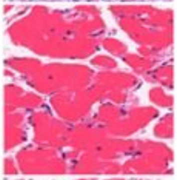	☐ TSCC<br>☑ Normal muscle tissue<br>☐ Not sure	☐ TSCC<br>☑ Normal muscle tissue<br>☐ Not sure	☐ TSCC<br>☑ Normal muscle tissue<br>☐ Not sure
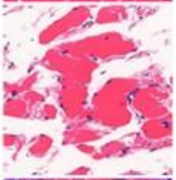	☐ TSCC<br>☑ Normal muscle tissue<br>☐ Not sure	☐ TSCC<br>☑ Normal muscle tissue<br>☐ Not sure	☐ TSCC<br>☑ Normal muscle tissue<br>☐ Not sure
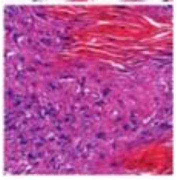	☑ TSCC<br>☐ Normal muscle tissue<br>☐ Not sure	☑ TSCC<br>☐ Normal muscle tissue<br>☐ Not sure	☑ TSCC<br>☐ Normal muscle tissue<br>☐ Not sure

TSCC Sample #8

Generated H&E images	Pathologist #1	Pathologist #2	Pathologist #3
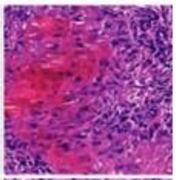	☑ TSCC<br>☐ Normal muscle tissue<br>☐ Not sure	☑ TSCC<br>☐ Normal muscle tissue<br>☐ Not sure	☑ TSCC<br>☐ Normal muscle tissue<br>☐ Not sure
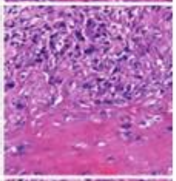	☑ TSCC<br>☐ Normal muscle tissue<br>☐ Not sure	☑ TSCC<br>☐ Normal muscle tissue<br>☐ Not sure	☑ TSCC<br>☐ Normal muscle tissue<br>☐ Not sure
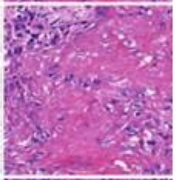	☑ TSCC<br>☐ Normal muscle tissue<br>☐ Not sure	☑ TSCC<br>☐ Normal muscle tissue<br>☐ Not sure	☑ TSCC<br>☐ Normal muscle tissue<br>☐ Not sure
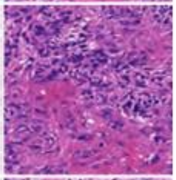	☑ TSCC<br>☐ Normal muscle tissue<br>☐ Not sure	☑ TSCC<br>☐ Normal muscle tissue<br>☐ Not sure	☑ TSCC<br>☐ Normal muscle tissue<br>☐ Not sure
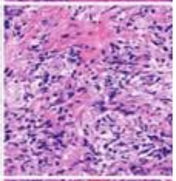	☑ TSCC<br>☐ Normal muscle tissue<br>☐ Not sure	☐ TSCC<br>☐ Normal muscle tissue<br>☑ Not sure	☑ TSCC<br>☐ Normal muscle tissue<br>☐ Not sure
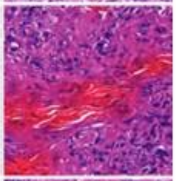	☑ TSCC<br>☐ Normal muscle tissue<br>☐ Not sure	☑ TSCC<br>☐ Normal muscle tissue<br>☐ Not sure	☑ TSCC<br>☐ Normal muscle tissue<br>☐ Not sure
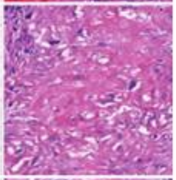	☑ TSCC<br>☐ Normal muscle tissue<br>☐ Not sure	☑ TSCC<br>☐ Normal muscle tissue<br>☐ Not sure	☑ TSCC<br>☐ Normal muscle tissue<br>☐ Not sure
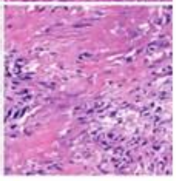	☑ TSCC<br>☐ Normal muscle tissue<br>☐ Not sure	☑ TSCC<br>☐ Normal muscle tissue<br>☐ Not sure	☑ TSCC<br>☐ Normal muscle tissue<br>☐ Not sure

TSCC Sample #9

Generated H&E images	Pathologist #1	Pathologist #2	Pathologist #3
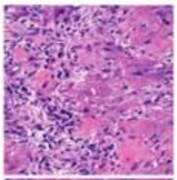	☑ TSCC<br>☐ Normal muscle tissue<br>☐ Not sure	☑ TSCC<br>☐ Normal muscle tissue<br>☐ Not sure	☑ TSCC<br>☐ Normal muscle tissue<br>☐ Not sure
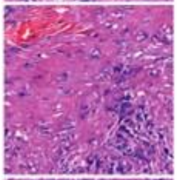	☑ TSCC<br>☐ Normal muscle tissue<br>☐ Not sure	☑ TSCC<br>☐ Normal muscle tissue<br>☐ Not sure	☑ TSCC<br>☐ Normal muscle tissue<br>☐ Not sure
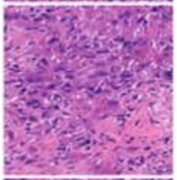	☑ TSCC<br>☐ Normal muscle tissue<br>☐ Not sure	☑ TSCC<br>☐ Normal muscle tissue<br>☐ Not sure	☑ TSCC<br>☐ Normal muscle tissue<br>☐ Not sure
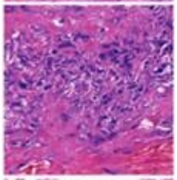	☑ TSCC<br>☐ Normal muscle tissue<br>☐ Not sure	☑ TSCC<br>☐ Normal muscle tissue<br>☐ Not sure	☑ TSCC<br>☐ Normal muscle tissue<br>☐ Not sure
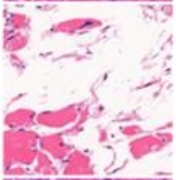	☐ TSCC<br>☑ Normal muscle tissue<br>☐ Not sure	☐ TSCC<br>☑ Normal muscle tissue<br>☐ Not sure	☐ TSCC<br>☑ Normal muscle tissue<br>☐ Not sure
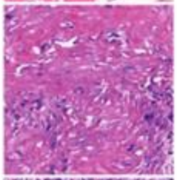	☑ TSCC<br>☐ Normal muscle tissue<br>☐ Not sure	☑ TSCC<br>☐ Normal muscle tissue<br>☐ Not sure	☑ TSCC<br>☐ Normal muscle tissue<br>☐ Not sure
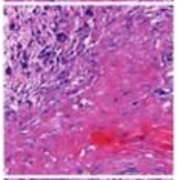	☑ TSCC<br>☐ Normal muscle tissue<br>☐ Not sure	☑ TSCC<br>☐ Normal muscle tissue<br>☐ Not sure	☑ TSCC<br>☐ Normal muscle tissue<br>☐ Not sure
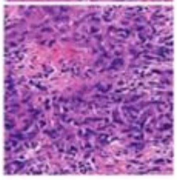	☑ TSCC<br>☐ Normal muscle tissue<br>☐ Not sure	☑ TSCC<br>☐ Normal muscle tissue<br>☐ Not sure	☑ TSCC<br>☐ Normal muscle tissue<br>☐ Not sure

TSCC Sample #10

Generated H&E images	Pathologist #1	Pathologist #2	Pathologist #3
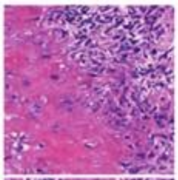	☑ TSCC<br>☐ Normal muscle tissue<br>☐ Not sure	☑ TSCC<br>☐ Normal muscle tissue<br>☐ Not sure	☑ TSCC<br>☐ Normal muscle tissue<br>☐ Not sure
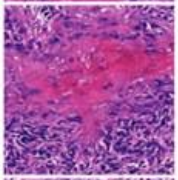	☑ TSCC<br>☐ Normal muscle tissue<br>☐ Not sure	☑ TSCC<br>☐ Normal muscle tissue<br>☐ Not sure	☑ TSCC<br>☐ Normal muscle tissue<br>☐ Not sure
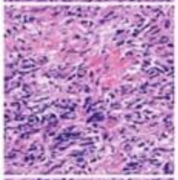	☑ TSCC<br>☐ Normal muscle tissue<br>☐ Not sure	☐ TSCC<br>☐ Normal muscle tissue<br>☑ Not sure	☑ TSCC<br>☐ Normal muscle tissue<br>☐ Not sure
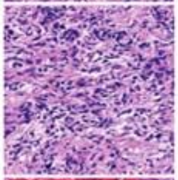	☑ TSCC<br>☐ Normal muscle tissue<br>☐ Not sure	☐ TSCC<br>☐ Normal muscle tissue<br>☑ Not sure	☑ TSCC<br>☐ Normal muscle tissue<br>☐ Not sure
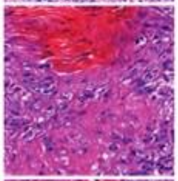	☑ TSCC<br>☐ Normal muscle tissue<br>☐ Not sure	☑ TSCC<br>☐ Normal muscle tissue<br>☐ Not sure	☑ TSCC<br>☐ Normal muscle tissue<br>☐ Not sure
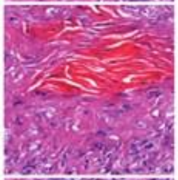	☑ TSCC<br>☐ Normal muscle tissue<br>☐ Not sure	☑ TSCC<br>☐ Normal muscle tissue<br>☐ Not sure	☑ TSCC<br>☐ Normal muscle tissue<br>☐ Not sure
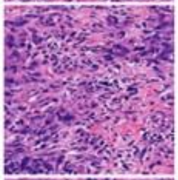	☑ TSCC<br>☐ Normal muscle tissue<br>☐ Not sure	☑ TSCC<br>☐ Normal muscle tissue<br>☐ Not sure	☑ TSCC<br>☐ Normal muscle tissue<br>☐ Not sure
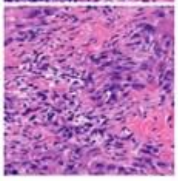	☑ TSCC<br>☐ Normal muscle tissue<br>☐ Not sure	☑ TSCC<br>☐ Normal muscle tissue<br>☐ Not sure	☑ TSCC<br>☐ Normal muscle tissue<br>☐ Not sure

TSCC Sample #11

Generated H&E images	Pathologist #1	Pathologist #2	Pathologist #3
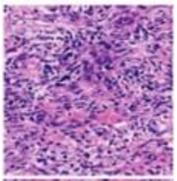	☑ TSCC<br>☐ Normal muscle tissue<br>☐ Not sure	☑ TSCC<br>☐ Normal muscle tissue<br>☐ Not sure	☑ TSCC<br>☐ Normal muscle tissue<br>☐ Not sure
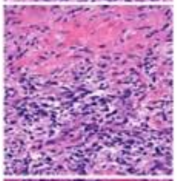	☑ TSCC<br>☐ Normal muscle tissue<br>☐ Not sure	☑ TSCC<br>☐ Normal muscle tissue<br>☐ Not sure	☑ TSCC<br>☐ Normal muscle tissue<br>☐ Not sure
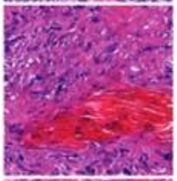	☑ TSCC<br>☐ Normal muscle tissue<br>☐ Not sure	☑ TSCC<br>☐ Normal muscle tissue<br>☐ Not sure	☑ TSCC<br>☐ Normal muscle tissue<br>☐ Not sure
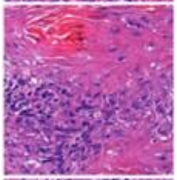	☑ TSCC<br>☐ Normal muscle tissue<br>☐ Not sure	☑ TSCC<br>☐ Normal muscle tissue<br>☐ Not sure	☑ TSCC<br>☐ Normal muscle tissue<br>☐ Not sure
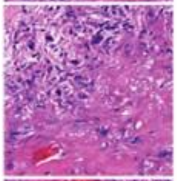	☑ TSCC<br>☐ Normal muscle tissue<br>☐ Not sure	☑ TSCC<br>☐ Normal muscle tissue<br>☐ Not sure	☑ TSCC<br>☐ Normal muscle tissue<br>☐ Not sure
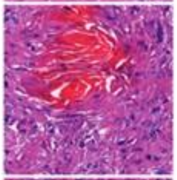	☑ TSCC<br>☐ Normal muscle tissue<br>☐ Not sure	☑ TSCC<br>☐ Normal muscle tissue<br>☐ Not sure	☑ TSCC<br>☐ Normal muscle tissue<br>☐ Not sure
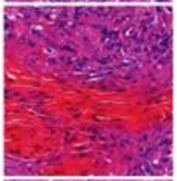	☑ TSCC<br>☐ Normal muscle tissue<br>☐ Not sure	☑ TSCC<br>☐ Normal muscle tissue<br>☐ Not sure	☑ TSCC<br>☐ Normal muscle tissue<br>☐ Not sure
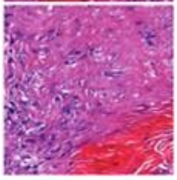	☑ TSCC<br>☐ Normal muscle tissue<br>☐ Not sure	☑ TSCC<br>☐ Normal muscle tissue<br>☐ Not sure	☑ TSCC<br>☐ Normal muscle tissue<br>☐ Not sure

TSCC Sample #12

Generated H&E images	Pathologist #1	Pathologist #2	Pathologist #3
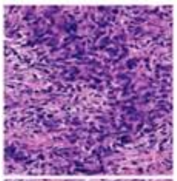	☑ TSCC<br>☐ Normal muscle tissue<br>☐ Not sure	☑ TSCC<br>☐ Normal muscle tissue<br>☐ Not sure	☑ TSCC<br>☐ Normal muscle tissue<br>☐ Not sure
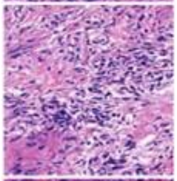	☑ TSCC<br>☐ Normal muscle tissue<br>☐ Not sure	☐ TSCC<br>☐ Normal muscle tissue<br>☑ Not sure	☑ TSCC<br>☐ Normal muscle tissue<br>☐ Not sure
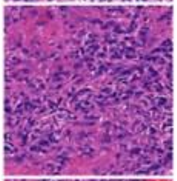	☑ TSCC<br>☐ Normal muscle tissue<br>☐ Not sure	☑ TSCC<br>☐ Normal muscle tissue<br>☐ Not sure	☑ TSCC<br>☐ Normal muscle tissue<br>☐ Not sure
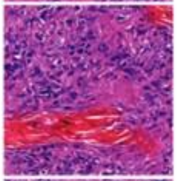	☑ TSCC<br>☐ Normal muscle tissue<br>☐ Not sure	☑ TSCC<br>☐ Normal muscle tissue<br>☐ Not sure	☑ TSCC<br>☐ Normal muscle tissue<br>☐ Not sure
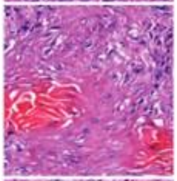	☑ TSCC<br>☐ Normal muscle tissue<br>☐ Not sure	☑ TSCC<br>☐ Normal muscle tissue<br>☐ Not sure	☑ TSCC<br>☐ Normal muscle tissue<br>☐ Not sure
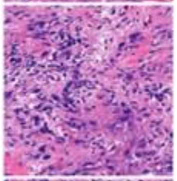	☑ TSCC<br>☐ Normal muscle tissue<br>☐ Not sure	☑ TSCC<br>☐ Normal muscle tissue<br>☐ Not sure	☑ TSCC<br>☐ Normal muscle tissue<br>☐ Not sure
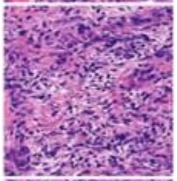	☑ TSCC<br>☐ Normal muscle tissue<br>☐ Not sure	☑ TSCC<br>☐ Normal muscle tissue<br>☐ Not sure	☑ TSCC<br>☐ Normal muscle tissue<br>☐ Not sure
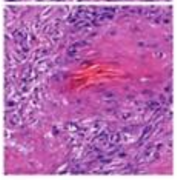	☑ TSCC<br>☐ Normal muscle tissue<br>☐ Not sure	☑ TSCC<br>☐ Normal muscle tissue<br>☐ Not sure	☑ TSCC<br>☐ Normal muscle tissue<br>☐ Not sure

TSCC Sample #13

Generated H&E images	Pathologist #1	Pathologist #2	Pathologist #3
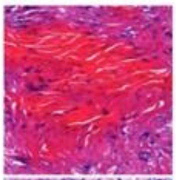	☑ TSCC<br>☐ Normal muscle tissue<br>☐ Not sure	☑ TSCC<br>☐ Normal muscle tissue<br>☐ Not sure	☐ TSCC<br>☐ Normal muscle tissue<br>☑ Not sure
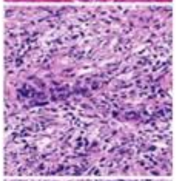	☑ TSCC<br>☐ Normal muscle tissue<br>☐ Not sure	☐ TSCC<br>☐ Normal muscle tissue<br>☑ Not sure	☑ TSCC<br>☐ Normal muscle tissue<br>☐ Not sure
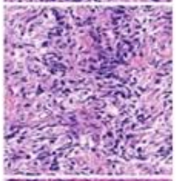	☑ TSCC<br>☐ Normal muscle tissue<br>☐ Not sure	☐ TSCC<br>☐ Normal muscle tissue<br>☑ Not sure	☑ TSCC<br>☐ Normal muscle tissue<br>☐ Not sure
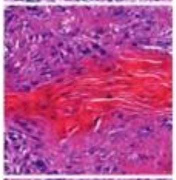	☑ TSCC<br>☐ Normal muscle tissue<br>☐ Not sure	☑ TSCC<br>☐ Normal muscle tissue<br>☐ Not sure	☑ TSCC<br>☐ Normal muscle tissue<br>☐ Not sure
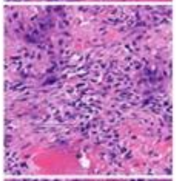	☑ TSCC<br>☐ Normal muscle tissue<br>☐ Not sure	☑ TSCC<br>☐ Normal muscle tissue<br>☐ Not sure	☑ TSCC<br>☐ Normal muscle tissue<br>☐ Not sure
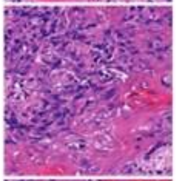	☑ TSCC<br>☐ Normal muscle tissue<br>☐ Not sure	☑ TSCC<br>☐ Normal muscle tissue<br>☐ Not sure	☑ TSCC<br>☐ Normal muscle tissue<br>☐ Not sure
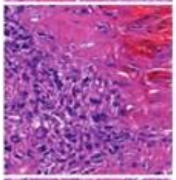	☑ TSCC<br>☐ Normal muscle tissue<br>☐ Not sure	☑ TSCC<br>☐ Normal muscle tissue<br>☐ Not sure	☑ TSCC<br>☐ Normal muscle tissue<br>☐ Not sure
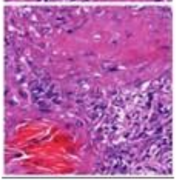	☑ TSCC<br>☐ Normal muscle tissue<br>☐ Not sure	☑ TSCC<br>☐ Normal muscle tissue<br>☐ Not sure	☑ TSCC<br>☐ Normal muscle tissue<br>☐ Not sure

TSCC Sample #14

Generated H&E images	Pathologist #1	Pathologist #2	Pathologist #3
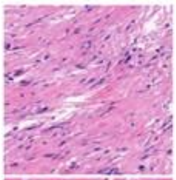	☐ TSCC<br>☑ Normal muscle tissue<br>☐ Not sure	☐ TSCC<br>☐ Normal muscle tissue<br>☑ Not sure	☐ TSCC<br>☑ Normal muscle tissue<br>☐ Not sure
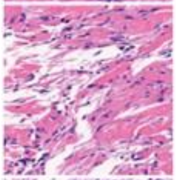	☐ TSCC<br>☑ Normal muscle tissue<br>☐ Not sure	☐ TSCC<br>☐ Normal muscle tissue<br>☑ Not sure	☐ TSCC<br>☑ Normal muscle tissue<br>☐ Not sure
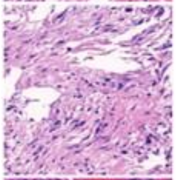	☑ TSCC<br>☐ Normal muscle tissue<br>☐ Not sure	☐ TSCC<br>☐ Normal muscle tissue<br>☑ Not sure	☑ TSCC<br>☐ Normal muscle tissue<br>☐ Not sure
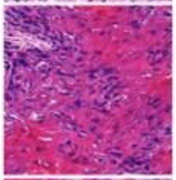	☑ TSCC<br>☐ Normal muscle tissue<br>☐ Not sure	☑ TSCC<br>☐ Normal muscle tissue<br>☐ Not sure	☑ TSCC<br>☐ Normal muscle tissue<br>☐ Not sure
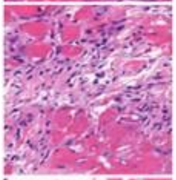	☑ TSCC<br>☐ Normal muscle tissue<br>☐ Not sure	☐ TSCC<br>☐ Normal muscle tissue<br>☑ Not sure	☐ TSCC<br>☐ Normal muscle tissue<br>☑ Not sure
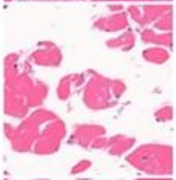	☐ TSCC<br>☑ Normal muscle tissue<br>☐ Not sure	☐ TSCC<br>☑ Normal muscle tissue<br>☐ Not sure	☐ TSCC<br>☑ Normal muscle tissue<br>☐ Not sure
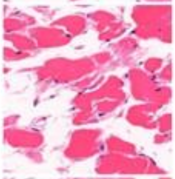	☐ TSCC<br>☑ Normal muscle tissue<br>☐ Not sure	☐ TSCC<br>☑ Normal muscle tissue<br>☐ Not sure	☐ TSCC<br>☑ Normal muscle tissue<br>☐ Not sure
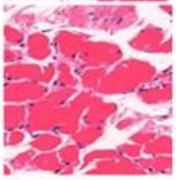	☐ TSCC<br>☑ Normal muscle tissue<br>☐ Not sure	☐ TSCC<br>☑ Normal muscle tissue<br>☐ Not sure	☐ TSCC<br>☑ Normal muscle tissue<br>☐ Not sure

TSCC Sample #15

Generated H&E images	Pathologist #1	Pathologist #2	Pathologist #3
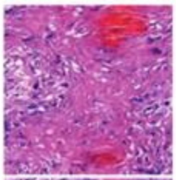	☑ TSCC<br>☐ Normal muscle tissue<br>☐ Not sure	☑ TSCC<br>☐ Normal muscle tissue<br>☐ Not sure	☑ TSCC<br>☐ Normal muscle tissue<br>☐ Not sure
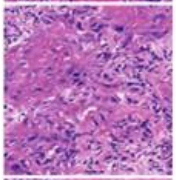	☑ TSCC<br>☐ Normal muscle tissue<br>☐ Not sure	☑ TSCC<br>☐ Normal muscle tissue<br>☐ Not sure	☑ TSCC<br>☐ Normal muscle tissue<br>☐ Not sure
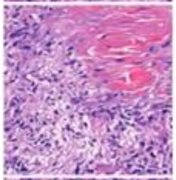	☑ TSCC<br>☐ Normal muscle tissue<br>☐ Not sure	☑ TSCC<br>☐ Normal muscle tissue<br>☐ Not sure	☑ TSCC<br>☐ Normal muscle tissue<br>☐ Not sure
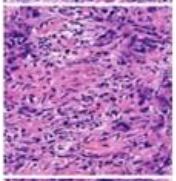	☑ TSCC<br>☐ Normal muscle tissue<br>☐ Not sure	☑ TSCC<br>☐ Normal muscle tissue<br>☐ Not sure	☑ TSCC<br>☐ Normal muscle tissue<br>☐ Not sure
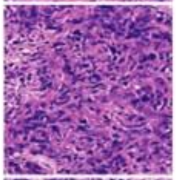	☑ TSCC<br>☐ Normal muscle tissue<br>☐ Not sure	☑ TSCC<br>☐ Normal muscle tissue<br>☐ Not sure	☑ TSCC<br>☐ Normal muscle tissue<br>☐ Not sure
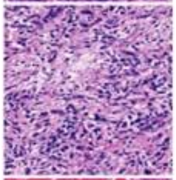	☑ TSCC<br>☐ Normal muscle tissue<br>☐ Not sure	☐ TSCC<br>☐ Normal muscle tissue<br>☑ Not sure	☑ TSCC<br>☐ Normal muscle tissue<br>☐ Not sure
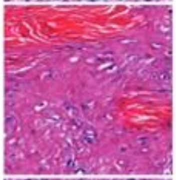	☑ TSCC<br>☐ Normal muscle tissue<br>☐ Not sure	☑ TSCC<br>☐ Normal muscle tissue<br>☐ Not sure	☑ TSCC<br>☐ Normal muscle tissue<br>☐ Not sure
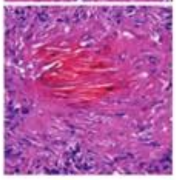	☑ TSCC<br>☐ Normal muscle tissue<br>☐ Not sure	☑ TSCC<br>☐ Normal muscle tissue<br>☐ Not sure	☑ TSCC<br>☐ Normal muscle tissue<br>☐ Not sure

Normal Sample #1

Generated H&E images	Pathologist #1	Pathologist #2	Pathologist #3
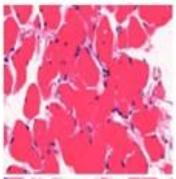	☐ TSCC<br>☑ Normal muscle tissue<br>☐ Not sure	☐ TSCC<br>☑ Normal muscle tissue<br>☐ Not sure	☐ TSCC<br>☑ Normal muscle tissue<br>☐ Not sure
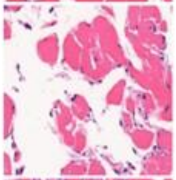	☐ TSCC<br>☑ Normal muscle tissue<br>☐ Not sure	☐ TSCC<br>☑ Normal muscle tissue<br>☐ Not sure	☐ TSCC<br>☑ Normal muscle tissue<br>☐ Not sure
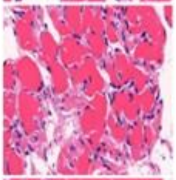	☐ TSCC<br>☑ Normal muscle tissue<br>☐ Not sure	☐ TSCC<br>☑ Normal muscle tissue<br>☐ Not sure	☐ TSCC<br>☑ Normal muscle tissue<br>☐ Not sure
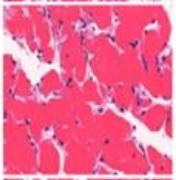	☐ TSCC<br>☑ Normal muscle tissue<br>☐ Not sure	☐ TSCC<br>☑ Normal muscle tissue<br>☐ Not sure	☐ TSCC<br>☑ Normal muscle tissue<br>☐ Not sure
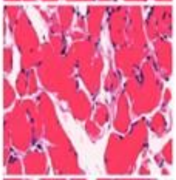	☐ TSCC<br>☑ Normal muscle tissue<br>☐ Not sure	☐ TSCC<br>☑ Normal muscle tissue<br>☐ Not sure	☐ TSCC<br>☑ Normal muscle tissue<br>☐ Not sure
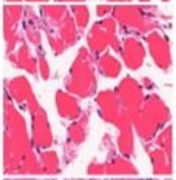	☐ TSCC<br>☑ Normal muscle tissue<br>☐ Not sure	☐ TSCC<br>☑ Normal muscle tissue<br>☐ Not sure	☐ TSCC<br>☑ Normal muscle tissue<br>☐ Not sure
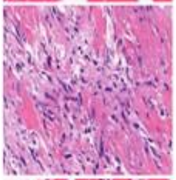	☐ TSCC<br>☐ Normal muscle tissue<br>☑ Not sure	☐ TSCC<br>☐ Normal muscle tissue<br>☑ Not sure	☑ TSCC<br>☐ Normal muscle tissue<br>☐ Not sure
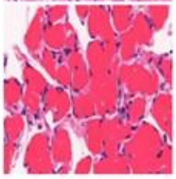	☐ TSCC<br>☑ Normal muscle tissue<br>☐ Not sure	☐ TSCC<br>☑ Normal muscle tissue<br>☐ Not sure	☐ TSCC<br>☑ Normal muscle tissue<br>☐ Not sure

Normal Sample #2

Generated H&E images	Pathologist #1	Pathologist #2	Pathologist #3
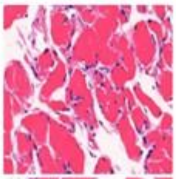	☐ TSCC<br>☑ Normal muscle tissue<br>☐ Not sure	☐ TSCC<br>☑ Normal muscle tissue<br>☐ Not sure	☐ TSCC<br>☑ Normal muscle tissue<br>☐ Not sure
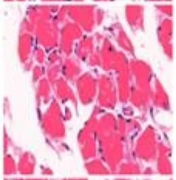	☐ TSCC<br>☑ Normal muscle tissue<br>☐ Not sure	☐ TSCC<br>☑ Normal muscle tissue<br>☐ Not sure	☐ TSCC<br>☑ Normal muscle tissue<br>☐ Not sure
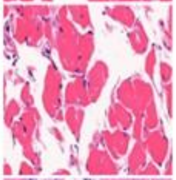	☐ TSCC<br>☑ Normal muscle tissue<br>☐ Not sure	☐ TSCC<br>☑ Normal muscle tissue<br>☐ Not sure	☐ TSCC<br>☑ Normal muscle tissue<br>☐ Not sure
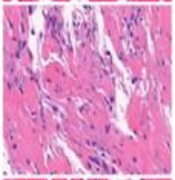	☐ TSCC<br>☑ Normal muscle tissue<br>☐ Not sure	☐ TSCC<br>☐ Normal muscle tissue<br>☑ Not sure	☐ TSCC<br>☑ Normal muscle tissue<br>☐ Not sure
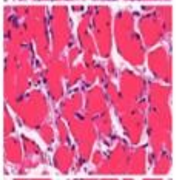	☐ TSCC<br>☑ Normal muscle tissue<br>☐ Not sure	☐ TSCC<br>☑ Normal muscle tissue<br>☐ Not sure	☐ TSCC<br>☑ Normal muscle tissue<br>☐ Not sure
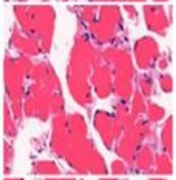	☐ TSCC<br>☑ Normal muscle tissue<br>☐ Not sure	☐ TSCC<br>☑ Normal muscle tissue<br>☐ Not sure	☐ TSCC<br>☑ Normal muscle tissue<br>☐ Not sure
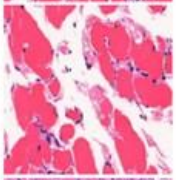	☐ TSCC<br>☑ Normal muscle tissue<br>☐ Not sure	☐ TSCC<br>☑ Normal muscle tissue<br>☐ Not sure	☐ TSCC<br>☑ Normal muscle tissue<br>☐ Not sure
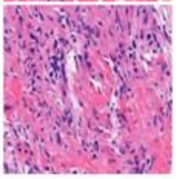	☑ TSCC<br>☐ Normal muscle tissue<br>☐ Not sure	☐ TSCC<br>☐ Normal muscle tissue<br>☑ Not sure	☑ TSCC<br>☐ Normal muscle tissue<br>☐ Not sure

Normal Sample #3

Generated H&E images	Pathologist #1	Pathologist #2	Pathologist #3
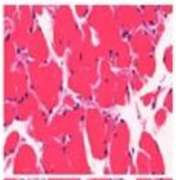	☐ TSCC<br>☑ Normal muscle tissue<br>☐ Not sure	☐ TSCC<br>☑ Normal muscle tissue<br>☐ Not sure	☐ TSCC<br>☑ Normal muscle tissue<br>☐ Not sure
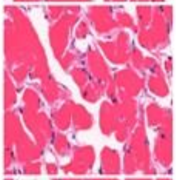	☐ TSCC<br>☑ Normal muscle tissue<br>☐ Not sure	☐ TSCC<br>☑ Normal muscle tissue<br>☐ Not sure	☐ TSCC<br>☑ Normal muscle tissue<br>☐ Not sure
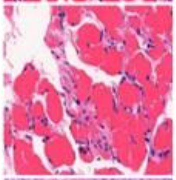	☐ TSCC<br>☑ Normal muscle tissue<br>☐ Not sure	☐ TSCC<br>☑ Normal muscle tissue<br>☐ Not sure	☐ TSCC<br>☑ Normal muscle tissue<br>☐ Not sure
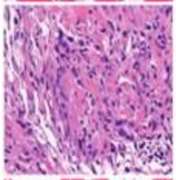	☑ TSCC<br>☐ Normal muscle tissue<br>☐ Not sure	☐ TSCC<br>☐ Normal muscle tissue<br>☑ Not sure	☑ TSCC<br>☐ Normal muscle tissue<br>☐ Not sure
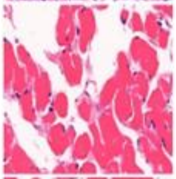	☐ TSCC<br>☑ Normal muscle tissue<br>☐ Not sure	☐ TSCC<br>☑ Normal muscle tissue<br>☐ Not sure	☐ TSCC<br>☑ Normal muscle tissue<br>☐ Not sure
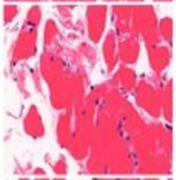	☐ TSCC<br>☑ Normal muscle tissue<br>☐ Not sure	☐ TSCC<br>☑ Normal muscle tissue<br>☐ Not sure	☐ TSCC<br>☑ Normal muscle tissue<br>☐ Not sure
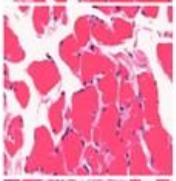	☐ TSCC<br>☑ Normal muscle tissue<br>☐ Not sure	☐ TSCC<br>☑ Normal muscle tissue<br>☐ Not sure	☐ TSCC<br>☑ Normal muscle tissue<br>☐ Not sure
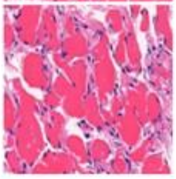	☐ TSCC<br>☑ Normal muscle tissue<br>☐ Not sure	☐ TSCC<br>☑ Normal muscle tissue<br>☐ Not sure	☐ TSCC<br>☑ Normal muscle tissue<br>☐ Not sure

Normal Sample #4

Generated H&E images	Pathologist #1	Pathologist #2	Pathologist #3
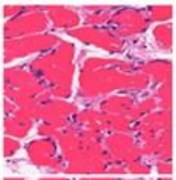	☐ TSCC<br>☑ Normal muscle tissue<br>☐ Not sure	☐ TSCC<br>☑ Normal muscle tissue<br>☐ Not sure	☐ TSCC<br>☑ Normal muscle tissue<br>☐ Not sure
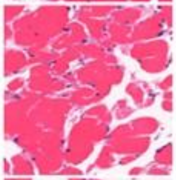	☐ TSCC<br>☑ Normal muscle tissue<br>☐ Not sure	☐ TSCC<br>☑ Normal muscle tissue<br>☐ Not sure	☐ TSCC<br>☑ Normal muscle tissue<br>☐ Not sure
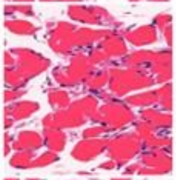	☐ TSCC<br>☑ Normal muscle tissue<br>☐ Not sure	☐ TSCC<br>☑ Normal muscle tissue<br>☐ Not sure	☐ TSCC<br>☑ Normal muscle tissue<br>☐ Not sure
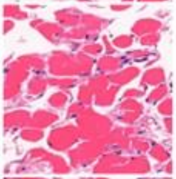	☐ TSCC<br>☑ Normal muscle tissue<br>☐ Not sure	☐ TSCC<br>☑ Normal muscle tissue<br>☐ Not sure	☐ TSCC<br>☑ Normal muscle tissue<br>☐ Not sure
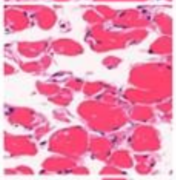	☐ TSCC<br>☑ Normal muscle tissue<br>☐ Not sure	☐ TSCC<br>☑ Normal muscle tissue<br>☐ Not sure	☐ TSCC<br>☑ Normal muscle tissue<br>☐ Not sure
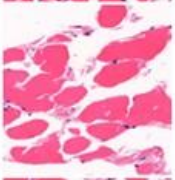	☐ TSCC<br>☑ Normal muscle tissue<br>☐ Not sure	☐ TSCC<br>☑ Normal muscle tissue<br>☐ Not sure	☐ TSCC<br>☑ Normal muscle tissue<br>☐ Not sure
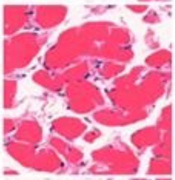	☐ TSCC<br>☑ Normal muscle tissue<br>☐ Not sure	☐ TSCC<br>☑ Normal muscle tissue<br>☐ Not sure	☐ TSCC<br>☑ Normal muscle tissue<br>☐ Not sure
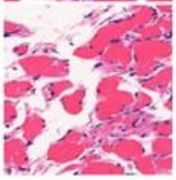	☐ TSCC<br>☑ Normal muscle tissue<br>☐ Not sure	☐ TSCC<br>☑ Normal muscle tissue<br>☐ Not sure	☐ TSCC<br>☑ Normal muscle tissue<br>☐ Not sure

Normal Sample #5

Generated H&E images	Pathologist #1	Pathologist #2	Pathologist #3
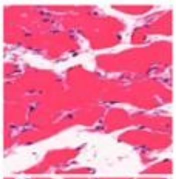	☐ TSCC<br>☑ Normal muscle tissue<br>☐ Not sure	☐ TSCC<br>☑ Normal muscle tissue<br>☐ Not sure	☐ TSCC<br>☑ Normal muscle tissue<br>☐ Not sure
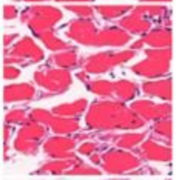	☐ TSCC<br>☑ Normal muscle tissue<br>☐ Not sure	☐ TSCC<br>☑ Normal muscle tissue<br>☐ Not sure	☐ TSCC<br>☑ Normal muscle tissue<br>☐ Not sure
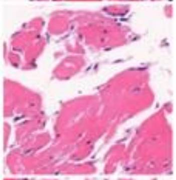	☐ TSCC<br>☑ Normal muscle tissue<br>☐ Not sure	☐ TSCC<br>☑ Normal muscle tissue<br>☐ Not sure	☐ TSCC<br>☑ Normal muscle tissue<br>☐ Not sure
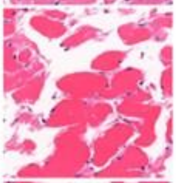	☐ TSCC<br>☑ Normal muscle tissue<br>☐ Not sure	☐ TSCC<br>☑ Normal muscle tissue<br>☐ Not sure	☐ TSCC<br>☑ Normal muscle tissue<br>☐ Not sure
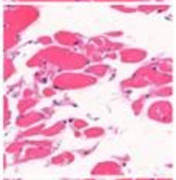	☐ TSCC<br>☑ Normal muscle tissue<br>☐ Not sure	☐ TSCC<br>☑ Normal muscle tissue<br>☐ Not sure	☐ TSCC<br>☑ Normal muscle tissue<br>☐ Not sure
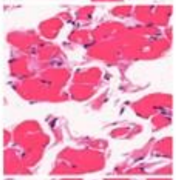	☐ TSCC<br>☑ Normal muscle tissue<br>☐ Not sure	☐ TSCC<br>☑ Normal muscle tissue<br>☐ Not sure	☐ TSCC<br>☑ Normal muscle tissue<br>☐ Not sure
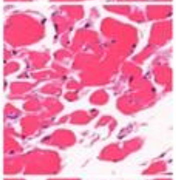	☐ TSCC<br>☑ Normal muscle tissue<br>☐ Not sure	☐ TSCC<br>☑ Normal muscle tissue<br>☐ Not sure	☐ TSCC<br>☑ Normal muscle tissue<br>☐ Not sure
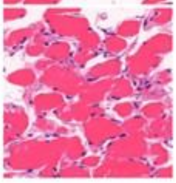	☐ TSCC<br>☑ Normal muscle tissue<br>☐ Not sure	☐ TSCC<br>☑ Normal muscle tissue<br>☐ Not sure	☐ TSCC<br>☑ Normal muscle tissue<br>☐ Not sure

Normal Sample #6

Generated H&E images	Pathologist #1	Pathologist #2	Pathologist #3
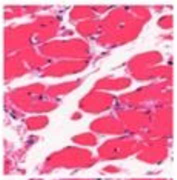	☐ TSCC<br>☑ Normal muscle tissue<br>☐ Not sure	☐ TSCC<br>☑ Normal muscle tissue<br>☐ Not sure	☐ TSCC<br>☑ Normal muscle tissue<br>☐ Not sure
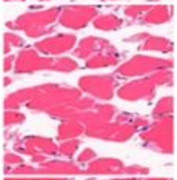	☐ TSCC<br>☑ Normal muscle tissue<br>☐ Not sure	☐ TSCC<br>☑ Normal muscle tissue<br>☐ Not sure	☐ TSCC<br>☑ Normal muscle tissue<br>☐ Not sure
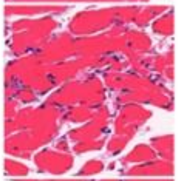	☐ TSCC<br>☑ Normal muscle tissue<br>☐ Not sure	☐ TSCC<br>☑ Normal muscle tissue<br>☐ Not sure	☐ TSCC<br>☑ Normal muscle tissue<br>☐ Not sure
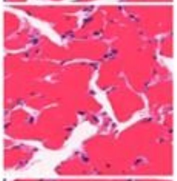	☐ TSCC<br>☑ Normal muscle tissue<br>☐ Not sure	☐ TSCC<br>☑ Normal muscle tissue<br>☐ Not sure	☐ TSCC<br>☑ Normal muscle tissue<br>☐ Not sure
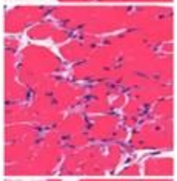	☐ TSCC<br>☑ Normal muscle tissue<br>☐ Not sure	☐ TSCC<br>☑ Normal muscle tissue<br>☐ Not sure	☐ TSCC<br>☑ Normal muscle tissue<br>☐ Not sure
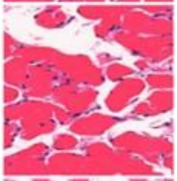	☐ TSCC<br>☑ Normal muscle tissue<br>☐ Not sure	☐ TSCC<br>☑ Normal muscle tissue<br>☐ Not sure	☐ TSCC<br>☑ Normal muscle tissue<br>☐ Not sure
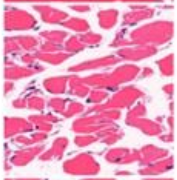	☐ TSCC<br>☑ Normal muscle tissue<br>☐ Not sure	☐ TSCC<br>☑ Normal muscle tissue<br>☐ Not sure	☐ TSCC<br>☑ Normal muscle tissue<br>☐ Not sure
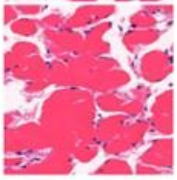	☐ TSCC<br>☑ Normal muscle tissue<br>☐ Not sure	☐ TSCC<br>☑ Normal muscle tissue<br>☐ Not sure	☐ TSCC<br>☑ Normal muscle tissue<br>☐ Not sure

Normal Sample #7

Generated H&E images	Pathologist #1	Pathologist #2	Pathologist #3
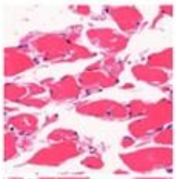	☐ TSCC<br>☑ Normal muscle tissue<br>☐ Not sure	☐ TSCC<br>☑ Normal muscle tissue<br>☐ Not sure	☐ TSCC<br>☑ Normal muscle tissue<br>☐ Not sure
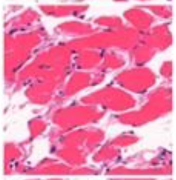	☐ TSCC<br>☑ Normal muscle tissue<br>☐ Not sure	☐ TSCC<br>☑ Normal muscle tissue<br>☐ Not sure	☐ TSCC<br>☑ Normal muscle tissue<br>☐ Not sure
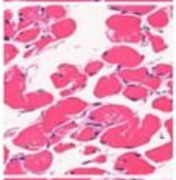	☐ TSCC<br>☑ Normal muscle tissue<br>☐ Not sure	☐ TSCC<br>☑ Normal muscle tissue<br>☐ Not sure	☐ TSCC<br>☑ Normal muscle tissue<br>☐ Not sure
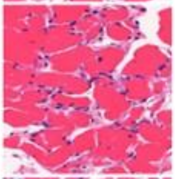	☐ TSCC<br>☑ Normal muscle tissue<br>☐ Not sure	☐ TSCC<br>☑ Normal muscle tissue<br>☐ Not sure	☐ TSCC<br>☑ Normal muscle tissue<br>☐ Not sure
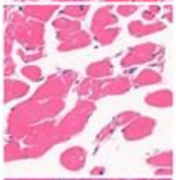	☐ TSCC<br>☑ Normal muscle tissue<br>☐ Not sure	☐ TSCC<br>☑ Normal muscle tissue<br>☐ Not sure	☐ TSCC<br>☑ Normal muscle tissue<br>☐ Not sure
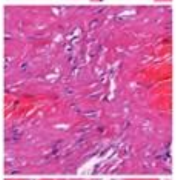	☑ TSCC<br>☐ Normal muscle tissue<br>☐ Not sure	☑ TSCC<br>☐ Normal muscle tissue<br>☐ Not sure	☐ TSCC<br>☐ Normal muscle tissue<br>☑ Not sure
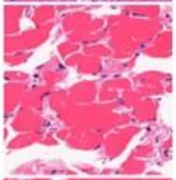	☐ TSCC<br>☑ Normal muscle tissue<br>☐ Not sure	☐ TSCC<br>☑ Normal muscle tissue<br>☐ Not sure	☐ TSCC<br>☑ Normal muscle tissue<br>☐ Not sure
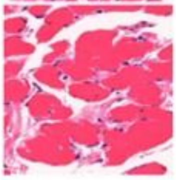	☐ TSCC<br>☑ Normal muscle tissue<br>☐ Not sure	☐ TSCC<br>☑ Normal muscle tissue<br>☐ Not sure	☐ TSCC<br>☑ Normal muscle tissue<br>☐ Not sure

Normal Sample #8

Generated H&E images	Pathologist #1	Pathologist #2	Pathologist #3
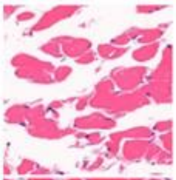	☐ TSCC<br>☑ Normal muscle tissue<br>☐ Not sure	☐ TSCC<br>☑ Normal muscle tissue<br>☐ Not sure	☐ TSCC<br>☑ Normal muscle tissue<br>☐ Not sure
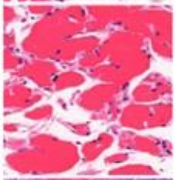	☐ TSCC<br>☑ Normal muscle tissue<br>☐ Not sure	☐ TSCC<br>☑ Normal muscle tissue<br>☐ Not sure	☐ TSCC<br>☑ Normal muscle tissue<br>☐ Not sure
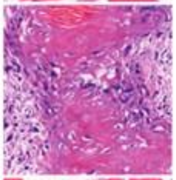	☑ TSCC<br>☐ Normal muscle tissue<br>☐ Not sure	☑ TSCC<br>☐ Normal muscle tissue<br>☐ Not sure	☑ TSCC<br>☐ Normal muscle tissue<br>☐ Not sure
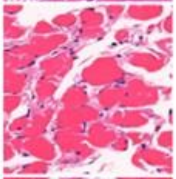	☐ TSCC<br>☑ Normal muscle tissue<br>☐ Not sure	☐ TSCC<br>☑ Normal muscle tissue<br>☐ Not sure	☐ TSCC<br>☑ Normal muscle tissue<br>☐ Not sure
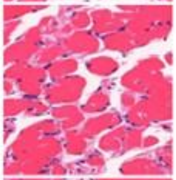	☐ TSCC<br>☑ Normal muscle tissue<br>☐ Not sure	☐ TSCC<br>☑ Normal muscle tissue<br>☐ Not sure	☐ TSCC<br>☑ Normal muscle tissue<br>☐ Not sure
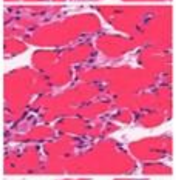	☐ TSCC<br>☑ Normal muscle tissue<br>☐ Not sure	☐ TSCC<br>☑ Normal muscle tissue<br>☐ Not sure	☐ TSCC<br>☑ Normal muscle tissue<br>☐ Not sure
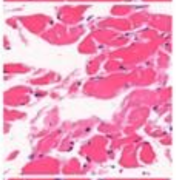	☐ TSCC<br>☑ Normal muscle tissue<br>☐ Not sure	☐ TSCC<br>☑ Normal muscle tissue<br>☐ Not sure	☐ TSCC<br>☑ Normal muscle tissue<br>☐ Not sure
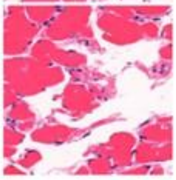	☐ TSCC<br>☑ Normal muscle tissue<br>☐ Not sure	☐ TSCC<br>☑ Normal muscle tissue<br>☐ Not sure	☐ TSCC<br>☑ Normal muscle tissue<br>☐ Not sure

Normal Sample #9

Generated H&E images	Pathologist #1	Pathologist #2	Pathologist #3
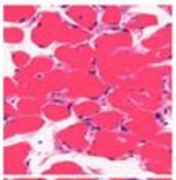	☐ TSCC<br>☑ Normal muscle tissue<br>☐ Not sure	☐ TSCC<br>☑ Normal muscle tissue<br>☐ Not sure	☐ TSCC<br>☑ Normal muscle tissue<br>☐ Not sure
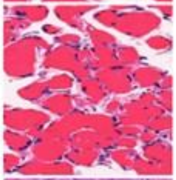	☐ TSCC<br>☑ Normal muscle tissue<br>☐ Not sure	☐ TSCC<br>☑ Normal muscle tissue<br>☐ Not sure	☐ TSCC<br>☑ Normal muscle tissue<br>☐ Not sure
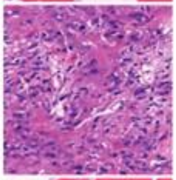	☑ TSCC<br>☐ Normal muscle tissue<br>☐ Not sure	☑ TSCC<br>☐ Normal muscle tissue<br>☐ Not sure	☑ TSCC<br>☐ Normal muscle tissue<br>☐ Not sure
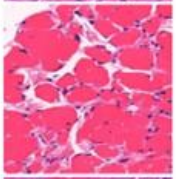	☐ TSCC<br>☑ Normal muscle tissue<br>☐ Not sure	☐ TSCC<br>☑ Normal muscle tissue<br>☐ Not sure	☐ TSCC<br>☑ Normal muscle tissue<br>☐ Not sure
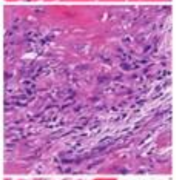	☑ TSCC<br>☐ Normal muscle tissue<br>☐ Not sure	☑ TSCC<br>☐ Normal muscle tissue<br>☐ Not sure	☑ TSCC<br>☐ Normal muscle tissue<br>☐ Not sure
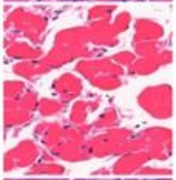	☐ TSCC<br>☑ Normal muscle tissue<br>☐ Not sure	☐ TSCC<br>☑ Normal muscle tissue<br>☐ Not sure	☐ TSCC<br>☑ Normal muscle tissue<br>☐ Not sure
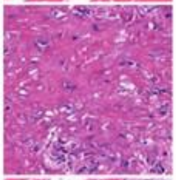	☑ TSCC<br>☐ Normal muscle tissue<br>☐ Not sure	☑ TSCC<br>☐ Normal muscle tissue<br>☐ Not sure	☑ TSCC<br>☐ Normal muscle tissue<br>☐ Not sure
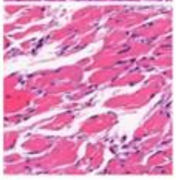	☐ TSCC<br>☑ Normal muscle tissue<br>☐ Not sure	☐ TSCC<br>☑ Normal muscle tissue<br>☐ Not sure	☐ TSCC<br>☑ Normal muscle tissue<br>☐ Not sure

Normal Sample #10

Generated H&E images	Pathologist #1	Pathologist #2	Pathologist #3
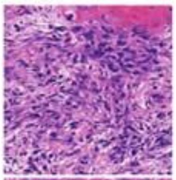	☑ TSCC<br>☐ Normal muscle tissue<br>☐ Not sure	☑ TSCC<br>☐ Normal muscle tissue<br>☐ Not sure	☑ TSCC<br>☐ Normal muscle tissue<br>☐ Not sure
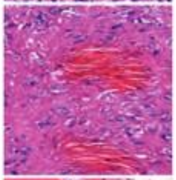	☑ TSCC<br>☐ Normal muscle tissue<br>☐ Not sure	☑ TSCC<br>☐ Normal muscle tissue<br>☐ Not sure	☑ TSCC<br>☐ Normal muscle tissue<br>☐ Not sure
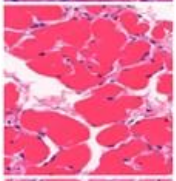	☐ TSCC<br>☑ Normal muscle tissue<br>☐ Not sure	☐ TSCC<br>☑ Normal muscle tissue<br>☐ Not sure	☐ TSCC<br>☑ Normal muscle tissue<br>☐ Not sure
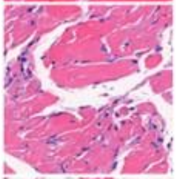	☐ TSCC<br>☑ Normal muscle tissue<br>☐ Not sure	☐ TSCC<br>☑ Normal muscle tissue<br>☐ Not sure	☐ TSCC<br>☑ Normal muscle tissue<br>☐ Not sure
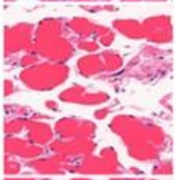	☐ TSCC<br>☑ Normal muscle tissue<br>☐ Not sure	☐ TSCC<br>☑ Normal muscle tissue<br>☐ Not sure	☐ TSCC<br>☑ Normal muscle tissue<br>☐ Not sure
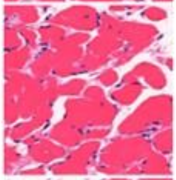	☐ TSCC<br>☑ Normal muscle tissue<br>☐ Not sure	☐ TSCC<br>☑ Normal muscle tissue<br>☐ Not sure	☐ TSCC<br>☑ Normal muscle tissue<br>☐ Not sure
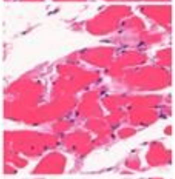	☐ TSCC<br>☑ Normal muscle tissue<br>☐ Not sure	☐ TSCC<br>☑ Normal muscle tissue<br>☐ Not sure	☐ TSCC<br>☑ Normal muscle tissue<br>☐ Not sure
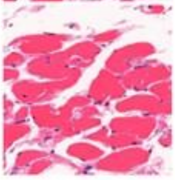	☐ TSCC<br>☑ Normal muscle tissue<br>☐ Not sure	☐ TSCC<br>☑ Normal muscle tissue<br>☐ Not sure	☐ TSCC<br>☑ Normal muscle tissue<br>☐ Not sure

Normal Sample #11

Generated H&E images	Pathologist #1	Pathologist #2	Pathologist #3
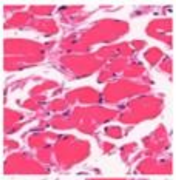	☐ TSCC<br>☑ Normal muscle tissue<br>☐ Not sure	☐ TSCC<br>☑ Normal muscle tissue<br>☐ Not sure	☐ TSCC<br>☑ Normal muscle tissue<br>☐ Not sure
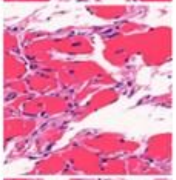	☐ TSCC<br>☑ Normal muscle tissue<br>☐ Not sure	☐ TSCC<br>☑ Normal muscle tissue<br>☐ Not sure	☐ TSCC<br>☑ Normal muscle tissue<br>☐ Not sure
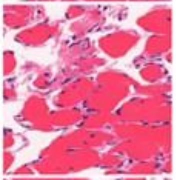	☐ TSCC<br>☑ Normal muscle tissue<br>☐ Not sure	☐ TSCC<br>☑ Normal muscle tissue<br>☐ Not sure	☐ TSCC<br>☑ Normal muscle tissue<br>☐ Not sure
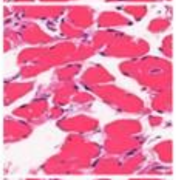	☐ TSCC<br>☑ Normal muscle tissue<br>☐ Not sure	☐ TSCC<br>☑ Normal muscle tissue<br>☐ Not sure	☐ TSCC<br>☑ Normal muscle tissue<br>☐ Not sure
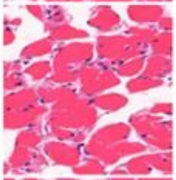	☐ TSCC<br>☑ Normal muscle tissue<br>☐ Not sure	☐ TSCC<br>☑ Normal muscle tissue<br>☐ Not sure	☐ TSCC<br>☑ Normal muscle tissue<br>☐ Not sure
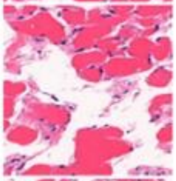	☐ TSCC<br>☑ Normal muscle tissue<br>☐ Not sure	☐ TSCC<br>☑ Normal muscle tissue<br>☐ Not sure	☐ TSCC<br>☑ Normal muscle tissue<br>☐ Not sure
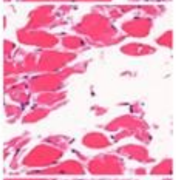	☐ TSCC<br>☑ Normal muscle tissue<br>☐ Not sure	☐ TSCC<br>☑ Normal muscle tissue<br>☐ Not sure	☐ TSCC<br>☑ Normal muscle tissue<br>☐ Not sure
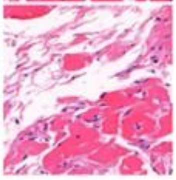	☐ TSCC<br>☑ Normal muscle tissue<br>☐ Not sure	☐ TSCC<br>☑ Normal muscle tissue<br>☐ Not sure	☐ TSCC<br>☑ Normal muscle tissue<br>☐ Not sure

Normal Sample #12

Generated H&E images	Pathologist #1	Pathologist #2	Pathologist #3
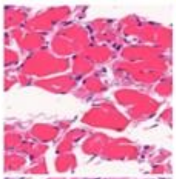	☐ TSCC<br>☑ Normal muscle tissue<br>☐ Not sure	☐ TSCC<br>☑ Normal muscle tissue<br>☐ Not sure	☐ TSCC<br>☑ Normal muscle tissue<br>☐ Not sure
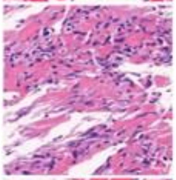	☑ TSCC<br>☐ Normal muscle tissue<br>☐ Not sure	☐ TSCC<br>☐ Normal muscle tissue<br>☑ Not sure	☑ TSCC<br>☐ Normal muscle tissue<br>☐ Not sure
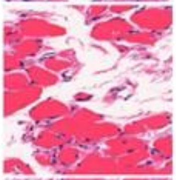	☐ TSCC<br>☑ Normal muscle tissue<br>☐ Not sure	☐ TSCC<br>☑ Normal muscle tissue<br>☐ Not sure	☐ TSCC<br>☑ Normal muscle tissue<br>☐ Not sure
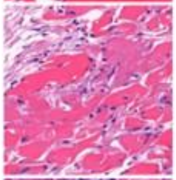	☐ TSCC<br>☑ Normal muscle tissue<br>☐ Not sure	☐ TSCC<br>☑ Normal muscle tissue<br>☐ Not sure	☐ TSCC<br>☑ Normal muscle tissue<br>☐ Not sure
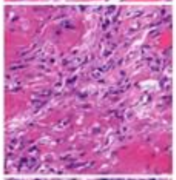	☑ TSCC<br>☐ Normal muscle tissue<br>☐ Not sure	☐ TSCC<br>☐ Normal muscle tissue<br>☑ Not sure	☑ TSCC<br>☐ Normal muscle tissue<br>☐ Not sure
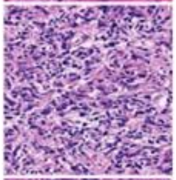	☑ TSCC<br>☐ Normal muscle tissue<br>☐ Not sure	☐ TSCC<br>☐ Normal muscle tissue<br>☑ Not sure	☑ TSCC<br>☐ Normal muscle tissue<br>☐ Not sure
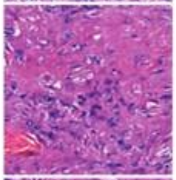	☑ TSCC<br>☐ Normal muscle tissue<br>☐ Not sure	☑ TSCC<br>☐ Normal muscle tissue<br>☐ Not sure	☑ TSCC<br>☐ Normal muscle tissue<br>☐ Not sure
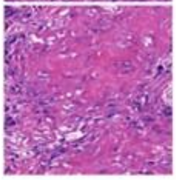	☑ TSCC<br>☐ Normal muscle tissue<br>☐ Not sure	☑ TSCC<br>☐ Normal muscle tissue<br>☐ Not sure	☑ TSCC<br>☐ Normal muscle tissue<br>☐ Not sure

Normal Sample #13

Generated H&E images	Pathologist #1	Pathologist #2	Pathologist #3
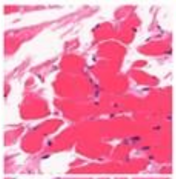	☐ TSCC<br>☑ Normal muscle tissue<br>☐ Not sure	☐ TSCC<br>☑ Normal muscle tissue<br>☐ Not sure	☐ TSCC<br>☑ Normal muscle tissue<br>☐ Not sure
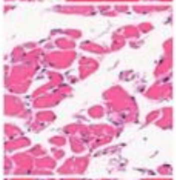	☐ TSCC<br>☑ Normal muscle tissue<br>☐ Not sure	☐ TSCC<br>☑ Normal muscle tissue<br>☐ Not sure	☐ TSCC<br>☑ Normal muscle tissue<br>☐ Not sure
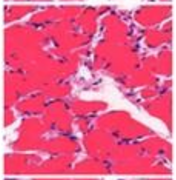	☐ TSCC<br>☑ Normal muscle tissue<br>☐ Not sure	☐ TSCC<br>☑ Normal muscle tissue<br>☐ Not sure	☐ TSCC<br>☑ Normal muscle tissue<br>☐ Not sure
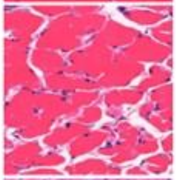	☐ TSCC<br>☑ Normal muscle tissue<br>☐ Not sure	☐ TSCC<br>☑ Normal muscle tissue<br>☐ Not sure	☐ TSCC<br>☑ Normal muscle tissue<br>☐ Not sure
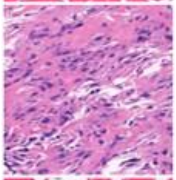	☑ TSCC<br>☐ Normal muscle tissue<br>☐ Not sure	☐ TSCC<br>☐ Normal muscle tissue<br>☑ Not sure	☑ TSCC<br>☐ Normal muscle tissue<br>☐ Not sure
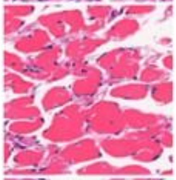	☐ TSCC<br>☑ Normal muscle tissue<br>☐ Not sure	☐ TSCC<br>☑ Normal muscle tissue<br>☐ Not sure	☐ TSCC<br>☑ Normal muscle tissue<br>☐ Not sure
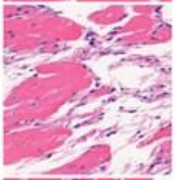	☐ TSCC<br>☑ Normal muscle tissue<br>☐ Not sure	☐ TSCC<br>☐ Normal muscle tissue<br>☑ Not sure	☐ TSCC<br>☑ Normal muscle tissue<br>☐ Not sure
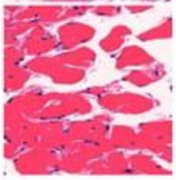	☐ TSCC<br>☑ Normal muscle tissue<br>☐ Not sure	☐ TSCC<br>☑ Normal muscle tissue<br>☐ Not sure	☐ TSCC<br>☑ Normal muscle tissue<br>☐ Not sure

Normal Sample #14

Generated H&E images	Pathologist #1	Pathologist #2	Pathologist #3
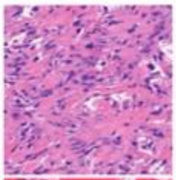	☑ TSCC<br>☐ Normal muscle tissue<br>☐ Not sure	☐ TSCC<br>☐ Normal muscle tissue<br>☑ Not sure	☑ TSCC<br>☐ Normal muscle tissue<br>☐ Not sure
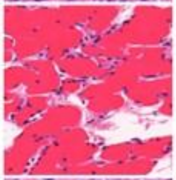	☐ TSCC<br>☑ Normal muscle tissue<br>☐ Not sure	☐ TSCC<br>☑ Normal muscle tissue<br>☐ Not sure	☐ TSCC<br>☑ Normal muscle tissue<br>☐ Not sure
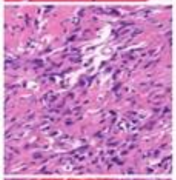	☑ TSCC<br>☐ Normal muscle tissue<br>☐ Not sure	☐ TSCC<br>☐ Normal muscle tissue<br>☑ Not sure	☑ TSCC<br>☐ Normal muscle tissue<br>☐ Not sure
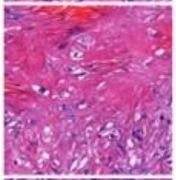	☑ TSCC<br>☐ Normal muscle tissue<br>☐ Not sure	☑ TSCC<br>☐ Normal muscle tissue<br>☐ Not sure	☑ TSCC<br>☐ Normal muscle tissue<br>☐ Not sure
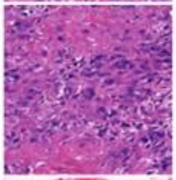	☑ TSCC<br>☐ Normal muscle tissue<br>☐ Not sure	☑ TSCC<br>☐ Normal muscle tissue<br>☐ Not sure	☑ TSCC<br>☐ Normal muscle tissue<br>☐ Not sure
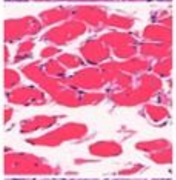	☐ TSCC<br>☑ Normal muscle tissue<br>☐ Not sure	☐ TSCC<br>☑ Normal muscle tissue<br>☐ Not sure	☐ TSCC<br>☑ Normal muscle tissue<br>☐ Not sure
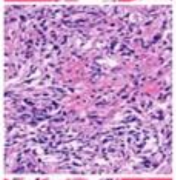	☑ TSCC<br>☐ Normal muscle tissue<br>☐ Not sure	☐ TSCC<br>☐ Normal muscle tissue<br>☑ Not sure	☑ TSCC<br>☐ Normal muscle tissue<br>☐ Not sure
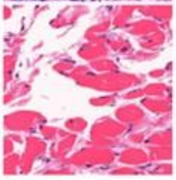	☐ TSCC<br>☑ Normal muscle tissue<br>☐ Not sure	☐ TSCC<br>☑ Normal muscle tissue<br>☐ Not sure	☐ TSCC<br>☑ Normal muscle tissue<br>☐ Not sure

Normal Sample #15

Generated H&E images	Pathologist #1	Pathologist #2	Pathologist #3
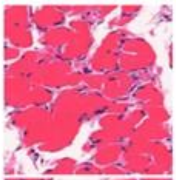	☐ TSCC<br>☑ Normal muscle tissue<br>☐ Not sure	☐ TSCC<br>☑ Normal muscle tissue<br>☐ Not sure	☐ TSCC<br>☑ Normal muscle tissue<br>☐ Not sure
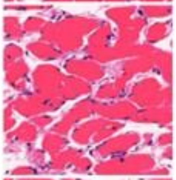	☐ TSCC<br>☑ Normal muscle tissue<br>☐ Not sure	☐ TSCC<br>☑ Normal muscle tissue<br>☐ Not sure	☐ TSCC<br>☑ Normal muscle tissue<br>☐ Not sure
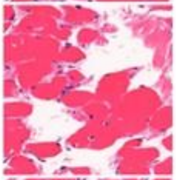	☐ TSCC<br>☑ Normal muscle tissue<br>☐ Not sure	☐ TSCC<br>☑ Normal muscle tissue<br>☐ Not sure	☐ TSCC<br>☑ Normal muscle tissue<br>☐ Not sure
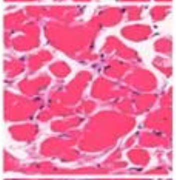	☐ TSCC<br>☑ Normal muscle tissue<br>☐ Not sure	☐ TSCC<br>☑ Normal muscle tissue<br>☐ Not sure	☐ TSCC<br>☑ Normal muscle tissue<br>☐ Not sure
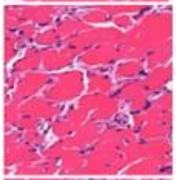	☐ TSCC<br>☑ Normal muscle tissue<br>☐ Not sure	☐ TSCC<br>☑ Normal muscle tissue<br>☐ Not sure	☐ TSCC<br>☑ Normal muscle tissue<br>☐ Not sure
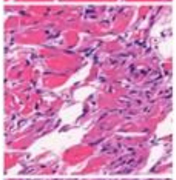	☐ TSCC<br>☑ Normal muscle tissue<br>☐ Not sure	☐ TSCC<br>☐ Normal muscle tissue<br>☑ Not sure	☐ TSCC<br>☑ Normal muscle tissue<br>☐ Not sure
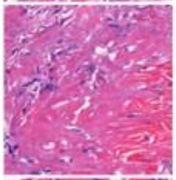	☑ TSCC<br>☐ Normal muscle tissue<br>☐ Not sure	☑ TSCC<br>☐ Normal muscle tissue<br>☐ Not sure	☑ TSCC<br>☐ Normal muscle tissue<br>☐ Not sure
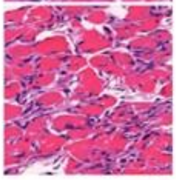	☐ TSCC<br>☑ Normal muscle tissue<br>☐ Not sure	☐ TSCC<br>☑ Normal muscle tissue<br>☐ Not sure	☐ TSCC<br>☑ Normal muscle tissue<br>☐ Not sure

Supplementary Table 3 Comparison of Model Performance in Classifying Raman Images on the Independent Test Set

Models	Pre-trained	TP	FP	FN	TN	ACC	F1	Recall	Precision	MCC
**ResNet18**	Yes	9	15	8	16	0.5208	0.5208	0.5208	0.5208	0.0436
	No	0	24	1	23	0.4792	0.4792	0.4792	0.4792	-0.1459
**ResNet 34**	Yes	23	1	19	5	0.5833	0.5833	0.5833	0.5833	0.2520
	No	15	9	13	11	0.5417	0.5417	0.5417	0.5417	0.0845
**ResNet 50**	Yes	20	4	11	13	0.6875	0.6875	0.6875	0.6875	0.3920
	No	0	24	0	24	0.5000	0.5000	0.5000	0.5000	0.0000
**ResNet 101**	Yes	19	5	12	12	0.6458	0.6458	0.6458	0.6458	0.3049
	No	14	10	10	14	0.5833	0.5833	0.5833	0.5833	0.1667
**ResNet 152**	Yes	14	10	6	18	0.6667	0.6667	0.6667	0.6667	0.3381
	No	1	23	1	23	0.5000	0.5000	0.5000	0.5000	0.0000
**Our model**		20	4	6	18	**0.7916**	**0.8000**	**0.8333**	**0.7692**	**0.5854**

Supplementary Table 4 The sample wise FID scores with different feature dimensions and independent manual review voting results

Label	Sample Number	FID Score from different feature dimensions				Independent Manual Review		
		64	192	768	2048	Normal	TSCC	Uncertain
Normal	1	8.11	25.26	1.14	319.48	0	22	2
	2	6.47	22.08	1.18	327.55	20	2	2
	3	6.93	22.62	1.00	293.33	21	2	1
	4	7.16	25.84	1.08	300.80	24	0	0
	5	11.49	48.63	1.12	304.73	24	0	0
	6	15.49	52.97	1.20	436.05	24	0	0
	7	6.75	25.93	0.95	313.02	21	2	1
	8	7.65	26.84	0.96	283.96	21	3	0
	9	18.24	63.61	1.43	365.93	15	9	0
	10	12.91	42.52	1.12	307.43	18	6	0
	11	6.93	25.85	0.84	263.06	24	0	0
	12	20.23	61.97	1.34	354.84	9	12	3
	13	9.07	32.88	1.04	306.38	20	2	2
	14	14.49	43.19	1.62	417.08	9	12	3
	15	13.41	43.17	1.34	308.45	20	3	1
TSCC	1	15.02	39.44	0.78	161.31	5	18	1
	2	21.41	51.02	0.86	200.57	6	18	0
	3	12.18	31.19	1.12	272.98	3	20	1
	4	16.57	43.12	1.19	230.54	6	18	0
	5	14.40	36.20	0.96	222.60	3	19	2
	6	7.14	22.98	1.28	344.67	0	23	1
	7	27.50	57.58	1.45	306.80	12	11	1
	8	17.10	42.17	0.95	165.27	0	23	1

	9	17.26	39.42	1.12	189.87	3	21	0
	10	15.67	36.69	0.83	146.50	0	22	2
	11	11.82	31.02	1.21	236.70	0	24	0
	12	16.00	38.24	0.95	206.90	0	23	1
	13	20.70	67.08	1.15	233.09	0	21	3
	14	20.30	50.35	1.37	285.34	13	7	4
	15	13.58	38.66	1.45	302.20	0	23	1
